# Nanoparticle-based modulation of CD4^+^ T cell effector and helper functions enhances adoptive immunotherapy

**DOI:** 10.1038/s41467-022-33597-y

**Published:** 2022-10-14

**Authors:** Ariel Isser, Aliyah B. Silver, Hawley C. Pruitt, Michal Mass, Emma H. Elias, Gohta Aihara, Si-Sim Kang, Niklas Bachmann, Ying-Yu Chen, Elissa K. Leonard, Joan G. Bieler, Worarat Chaisawangwong, Joseph Choy, Sydney R. Shannon, Sharon Gerecht, Jeffrey S. Weber, Jamie B. Spangler, Jonathan P. Schneck

**Affiliations:** 1 Department of Biomedical Engineering, Johns Hopkins University School of Medicine, Baltimore, MD 21287 USA; 2 Johns Hopkins Translational ImmunoEngineering Center, Johns Hopkins University School of Medicine, Baltimore, MD 21287 USA; 3 Department of Molecular Microbiology and Immunology, Johns Hopkins University Bloomberg School of Public Health, Baltimore, MD 21287 USA; 4 Translational Tissue Engineering Center, Johns Hopkins University School of Medicine, Baltimore, MD 21287 USA; 5 Department of Chemical and Biomolecular Engineering, Johns Hopkins University Whiting School of Engineering, Baltimore, MD 21287 USA; 6 Institute for NanoBioTechnology, Johns Hopkins University Whiting School of Engineering, Baltimore, MD 21287 USA; 7 Department of Biology, Johns Hopkins University Krieger School of Arts and Sciences, Baltimore, MD 21287 USA; 8 Department of Pathology, Johns Hopkins University School of Medicine, Baltimore, MD 21287 USA; 9 Department of Materials Science and Engineering, Johns Hopkins University Whiting School of Engineering, Baltimore, MD 21287 USA; 10 Department of Oncology, Johns Hopkins University School of Medicine, Baltimore, MD 21287 USA; 11 Laura and Isaac Perlmutter Cancer Center, NYU Langone Health, New York, NY 10016 USA; 12 Department of Ophthalmology, Wilmer Eye Institute, Johns Hopkins University School of Medicine, Baltimore, MD 21287 USA; 13 Bloomberg~Kimmel Institute for Cancer Immunotherapy, Sidney Kimmel Comprehensive Cancer Center, Johns Hopkins University School of Medicine, Baltimore, MD 21287 USA; 14 Institute for Cell Engineering, Johns Hopkins University School of Medicine, Baltimore, MD 21287 USA

**Keywords:** Nanoparticles, Applied immunology, Cancer immunotherapy, Lymphocyte activation

## Abstract

Helper (CD4^+^) T cells perform direct therapeutic functions and augment responses of cells such as cytotoxic (CD8^+^) T cells against a wide variety of diseases and pathogens. Nevertheless, inefficient synthetic technologies for expansion of antigen-specific CD4^+^ T cells hinders consistency and scalability of CD4^+^ T cell-based therapies, and complicates mechanistic studies. Here we describe a nanoparticle platform for ex vivo CD4^+^ T cell culture that mimics antigen presenting cells (APC) through display of major histocompatibility class II (MHC II) molecules. When combined with soluble co-stimulation signals, MHC II artificial APCs (aAPCs) expand cognate murine CD4^+^ T cells, including rare endogenous subsets, to induce potent effector functions in vitro and in vivo. Moreover, MHC II aAPCs provide help signals that enhance antitumor function of aAPC-activated CD8^+^ T cells in a mouse tumor model. Lastly, human leukocyte antigen class II-based aAPCs expand rare subsets of functional, antigen-specific human CD4^+^ T cells. Overall, MHC II aAPCs provide a promising approach for harnessing targeted CD4^+^ T cell responses.

## Abstract (web summary)

Adoptive cell therapies (ACT) hold promise for cancer immunotherapy, but optimization is still an ongoing process. Here the authors report CD4-targeted, nanoparticle-based artificial antigen-presenting cells that expand CD4^+^ T cells capable of lysing tumor cell lysis in vitro, and CD8^+^ T cells showing antitumor activity in a mouse melanoma model.

## Introduction

Clinical successes of adoptive cell transfer (ACT) therapies across a wide range of hematologic malignancies^1,2^ and solid tumors^3,4^ have propelled T cell therapies to the forefront of treatment options for a variety of cancers and other diseases. Despite their promise, some of the largest hurdles these therapies face in moving towards widespread translation are the associated time, costs, and complexities of ex vivo T cell expansion^5^, as well as the variability of the resulting clinical products.

A range of approaches have been developed for ex vivo expansion of tumor-specific T cells, including polyclonal T cell stimulation with plate- or bead-bound anti-CD3 (αCD3) antibodies or antigen-specific T cell stimulation with peptide-pulsed autologous antigen-presenting cells (APCs). To simultaneously address the lack of specificity of αCD3 stimulation as well as the manufacturing challenges and variability of donor-derived APCs, biomimetic artificial APCs (aAPCs) that include MHC proteins and co-stimulatory molecules have been produced^6^. Thus far, these synthetic platforms have focused almost exclusively on CD8^+^ T cells, whereas little progress has been made for CD4^+^ targeted technologies.

CD4^+^ T cells serve several critical functions in the antitumor immune response, including recognizing neoantigens that result from tumor-specific mutations^7,8^, recruiting and activating innate immune cells^9–11^, directly lysing MHC II positive tumor cells^12^, and relaying indispensable “help” signals to CD8^+^ T cells to enhance their antitumor function and memory formation^13^. A simplified system that modulates these functions could pave the way toward scalable, consistent CD4^+^ T cell or “helped” CD8^+^ T cell cancer therapies, while also providing mechanistic insight into CD4^+^ T cell tumor biology.

Herein, we describe a platform for antigen-specific CD4^+^ T cell expansion, consisting of iron-dextran nanoparticles coated with MHC II and co-stimulatory proteins. These MHC II aAPCs lead to the expansion of cognate murine CD4^+^ T cells that display high levels of effector cytokine production and demonstrate robust lytic capacity in vitro and in vivo. MHC II aAPCs also relay help signals from CD4^+^ T cells to tumor-specific CD8^+^ T cells, which, in turn, enhance CD8^+^ T cell cytokine production, memory formation, and in vitro and in vivo antitumor activity. Lastly, murine MHC II and human counterpart HLA II aAPCs can expand rare subsets of endogenous murine and human CD4^+^ T cells. Together, this work highlights a variety of applications of nanoparticle technologies for enrichment, expansion, and modulation of CD4^+^ T cell effector and helper functions.

## Results

### MHC II aAPCs stimulate functional antigen-specific murine CD4^+^ T cells

**Fig. 1:**
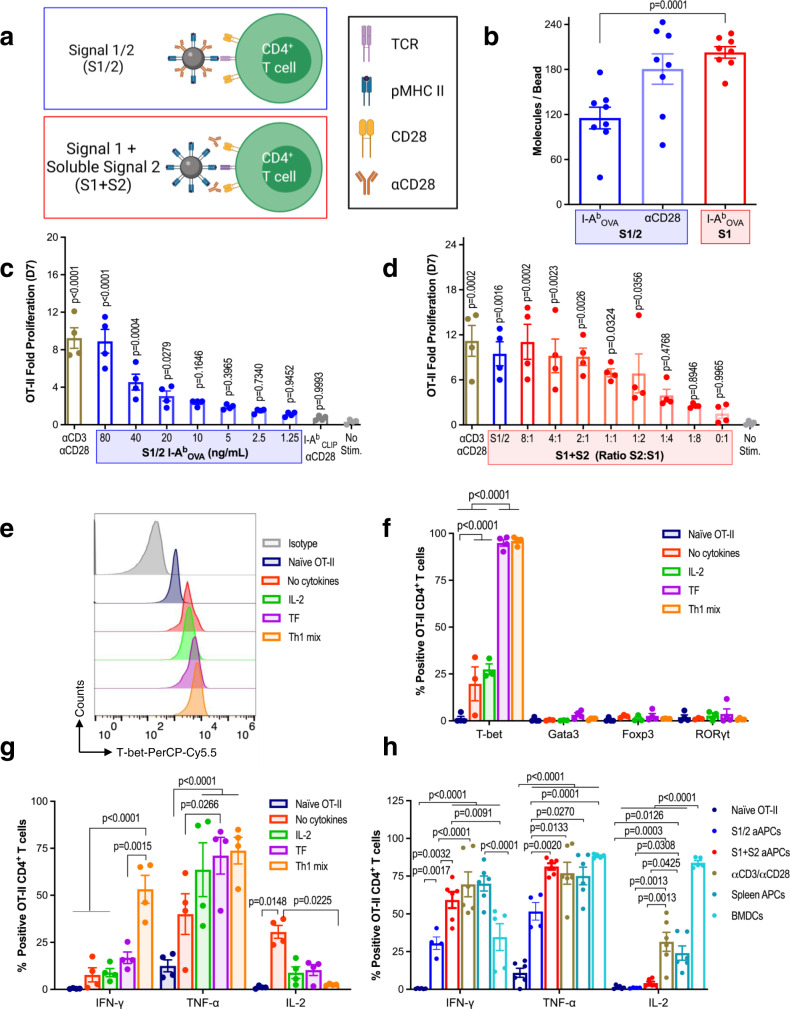
MHC II aAPCs stimulate functional antigen-specific murine CD4^+^ T cells. **a** Design of MHC II aAPCs with MHC class II molecules (MHC II) as Signal 1 (S1) and anti-CD28 antibodies (αCD28) as Signal 2 (S2). S2 is either attached to aAPCs (S1/2) or delivered solubly (S1 + S2). Created with BioRender.com. **b** Fluorescent quantification of I-A^b^_OVA_ and αCD28 conjugated to S1/2 and S1 aAPCs. **c** OT-II CD4^+^ T cell fold proliferation after 7 days of stimulation with I-A^b^_OVA_ S1/2 aAPCs compared to polyclonal αCD3/αCD28 or I-A^b^_CLIP_ aAPCs. **d** Day 7 OT-II fold proliferation following treatment with S1 aAPCs and titration of S2, compared to S1/2 or αCD3/αCD28 aAPCs. **e** Day 7 T-bet staining, **f** CD4^+^ lineage transcription factor staining, and **g** cytokine production of OT-II cells stimulated with S1/2 aAPCs in media containing: no cytokines, IL-2, T cell growth factor (TF) cytokine cocktail, or a Th1 mix (IL-2, IL-12p70, IFN-γ). **h** Day 7 cytokine production of OT-II cells stimulated with S1/2, S1 + S2, or αCD3/αCD28 aAPCs versus peptide-pulsed OT-II splenocytes or bone marrow-derived dendritic cells (BMDCs). Data in (**b**–**d**, **f**–**h**) represent the mean ± standard error of the mean (s.e.m.) from three or more independent experiments. **b** *n* = 8, **c**, **d** *n* = 4 mice, **f** *n* = 3 (no cytokines) or 4 mice (naïve OT-II, IL-2, TF, Th1 mix), **g** *n* = 4 mice, **h** *n* = 4 (S1/2), 5 (BMDCs), or 6 mice (Naïve, Spleen APCs, αCD3/αCD28, S1 + S2), analyzed using (**b**) *n* unpaired Student’s *t*-test, two-tailed, **c**, **d** A one-way ANOVA compared to “no stim.” with Dunnet’s multiple-comparisons test, or **f**–**h** a two-way ANOVA with Tukey’s multiple-comparisons test.

T cells require two signals to become activated: T cell receptor (TCR) stimulation known as signal 1 (S1) through cognate peptide-loaded MHC (pMHC) interactions and co-stimulation, termed signal 2 (S2), most commonly through the CD28 receptor. TCR-pMHC interactions tend to be a lower affinity for CD4^+^ T cells than for CD8^+^ T cells^14^. Based on this premise, we formulated two aAPC designs for ex vivo activation of antigen-specific murine CD4^+^ T cells: one that, similar to traditional MHC I aAPCs^6^, co-presents MHC II I-A^b^ proteins and anti-CD28 (αCD28) antibodies (S1/2) and a second that presents only I-A^b^ proteins, with the addition of soluble αCD28 (S1 + S2), to maximize MHC II valency on aAPCs (Fig. 1a). To synthesize the aAPCs, signals were conjugated to 200 nm iron oxide nanoparticles, a size which corresponds to the preformed TCR clusters found on naïve T cells^15^ and which we have previously shown is optimal for CD8^+^ T cell engagement^16^. Post fabrication, the aAPCs were ~300 nm in size (Supplementary Fig. 1a, b), with around 100 I-A^b^ molecules per S1/2 bead and 200 I-A^b^ molecules per S1 bead (Fig. 1b). Through titration of the S1/2 aAPCs into culture with TCR transgenic OT-II ovalbumin (OVA) specific CD4^+^ T cells, we found that a concentration of 80 ng/mL I-A^b^ loaded with the OVA_329-337_ peptide (I-A^b^_OVA_) led to a similar percentage of T cells dividing at day 3 (Supplementary Fig. 1c, d) and fold proliferation at day 7 (Fig. 1c) compared to control αCD3/αCD28 microbeads. The stimulation was antigen-specific, as I-A^b^ aAPCs loaded with an irrelevant CLIP_87-101_ peptide (I-A^b^_CLIP_) did not induce OT-II proliferation (Fig. 1c and Supplementary Fig. 1c, d). For S1 aAPCs, increasing the amount of soluble S2 added into the culture did not impact the percentage of cells dividing at day 3 (Supplementary Fig. 1e, f) but did increase fold proliferation at day 7 to levels similar to αCD3/αCD28 microbeads and S1/2 aAPCs (Fig. 1d). As certain CD4^+^ T cell subsets can either promote or inhibit antitumor responses^17^ (e.g., Th1 versus regulatory T cells), we next analyzed CD4^+^ T cell polarization, by activating OT-II CD4^+^ T cells with MHC II aAPCs in the presence of various cytokine mixes. In comparison to treatment with interleukin-2 (IL-2) only and no cytokines, a Th1 mix (IL-2, IL-12p70, and IFN-γ) led to higher T-bet expression (Fig. 1e, f) and IFN-γ production (Fig. 1g and Supplementary Fig. 1g), hallmark transcription factors and cytokines of the Th1 lineage, respectively. Interestingly, a standard T cell growth factor (TF) cytokine cocktail for CD8^+^ T cell culture^18^, led to a similar increase in T-bet levels but not IFN-γ production. We also compared the impact of different types of T cell stimulation on OT-II cell proliferation and function when cultured in the Th1 mix. We found that optimal doses of S1/2 and S1 + S2 aAPCs led to equivalent proliferation as αCD3/αCD28 microbeads, OT-II splenocytes pulsed with OVA_323-339_ peptide, or bone marrow-derived dendritic cells (BMDCs) (Supplementary Fig. 1h). Additionally, S1 + S2 aAPC stimulation led to IFN-γ production equivalent to peptide-pulsed splenocytes or αCD3/αCD28 microbeads, whereas cells cultured with S1/2 aAPCs or BMDCs produced less IFN-γ (Fig. 1h and Supplementary Fig. 1i). To assess the impact of signal density on aAPC-mediated OT-II proliferation and function, we mixed I-A^b^_OVA_ proteins at 1:1 and 1:3 molar ratios with isotype antibodies (S1/I) or bovine serum albumin (S1/B), detecting lower conjugation of I-A^b^ at higher ratios of these additional proteins (Supplementary Fig. 1j). We found that at equivalent doses of S1 (80 ng/mL), aAPCs with lower S1 density led to similar percentages of OT-II cells dividing at day 3 (Supplementary Fig. 1k), but lower overall proliferation at day 7 compared to higher density S1 or S1/2 aAPCs (Supplementary Fig. 1l). That said, the density of S1 did not impact OT-II function at day 7 (Supplementary Fig. 1m). We performed similar analyses using 4.5 μm S1/2 aAPCs that more closely mimic the size of endogenous APCs, observing no significant differences in OT-II proliferation or IFN-γ and TNF-α secretion compared to nano-aAPCs, but higher frequencies of IL-2 producing cells (Supplementary Fig. 1k–m). Together, these results demonstrate the robust expansion of functional, antigen-specific CD4^+^ T cell is achieved by both S1/2 and S1 + S2 MHC II aAPCs and that the extent of expansion is directly dependent upon S1 density.

### MHC II aAPCs expand rare murine CD4^+^ T cell subsets

**Fig. 2:**
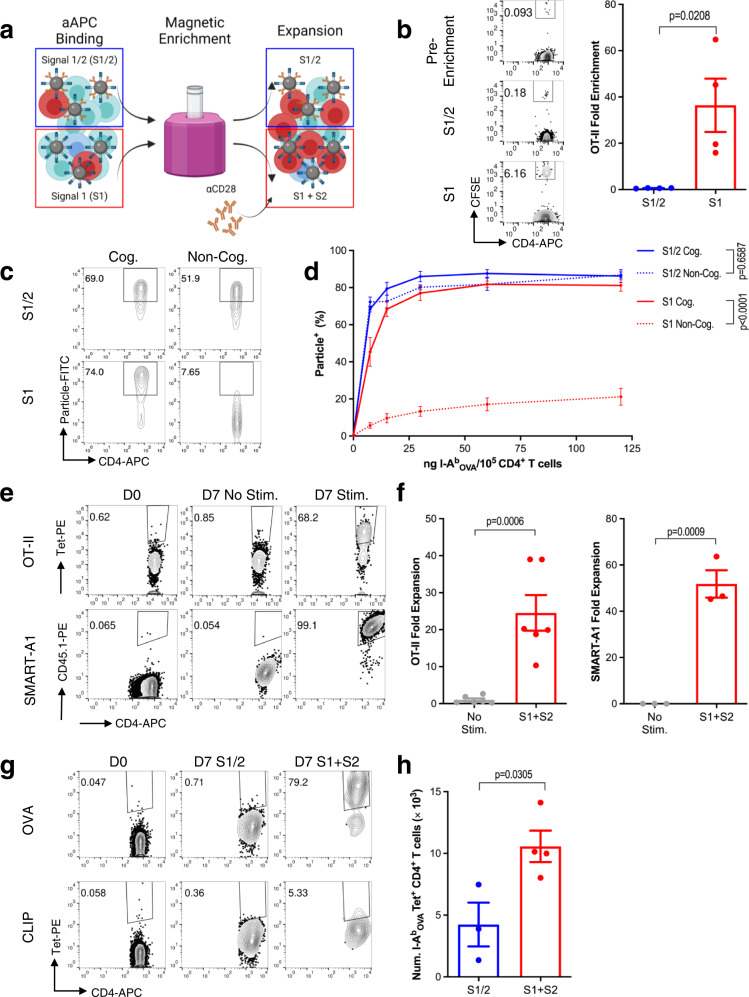
MHC II aAPCs expand rare murine CD4^+^ T cell subsets. **a** Schematic of magnetic enrichment of rare CD4^+^ T cells with aAPCs. Created with BioRender.com. **b** Representative flow plots (left) and fold enrichment (right) of OT-II cells diluted into a B6 background at a ratio of 1:1000 after magnetic enrichment with S1/2 or S1 aAPCs. **c** Representative flow plots and **d** percent of OT-II (cognate) and B6 (non-cognate) CD4^+^ T cells bound to particles after 2 h of incubation at 37 °C with S1/2 versus S1 aAPCs across a range of doses. **e** Representative flow plots and **f** fold expansion of OT-II and SMART-A1 cells diluted 1:1000 into a B6 background, as measured 7 days after S1 + S2 enrichment and expansion. **g** pMHC Tetramer staining and **h** quantified a number of I-A^b^_OVA_ CD4^+^ T cells 7 days after S1/2 or S1 + S2 enrichment and expansion. Data in **b**, **d**, **f**, **h** represent mean ± standard error of the mean (s.e.m.) from three or more independent experiments. **b** *n* = 4 mice, **d** *n* = 3 (S1/2) or 4 (S1) mice, (**f**.**1**) *n* = 6 mice, (**f**.**2**) *n* = 3 mice, **h** *n* = 3 (S1/2) or 4 (S1 + S2) mice, analyzed using an **b**, **f**, **h** unpaired Student’s *t*-test, two-tailed or **d** a two-way ANOVA with Tukey’s multiple-comparisons test.

To explore whether MHC II aAPCs could be used to expand rare antigen-specific CD4^+^ T cells, we employed an analogous approach to our previous work with murine^19,20^ and human^21^ CD8^+^ T cells, following a three-step process that includes aAPCs binding to T cells, magnetic enrichment of aAPC-bound T cells, and expansion of the enriched T cell product (Fig. 2a). For S1 aAPCs, excess soluble S2 was added to the enriched product to facilitate T cell expansion. To optimize the enrichment and expansion system, we diluted CFSE labeled OT-II CD4^+^ T cells at a ratio of 1:1000 into a background of C57BL/6 (B6) CD4^+^ T cells. Mimicking MHC II tetramer binding protocols^22^, we incubated the mixed cell population with aAPCs at 37 °C for 2 h, followed by magnetic enrichment of aAPC-bound cells using a 96-well plate magnet compatible with our 300 nm aAPCs^23^. Immediately post-enrichment, we found that S1 aAPCs led to significantly higher fold enrichment than S1/2 aAPCs (Fig. 2b). To understand why this was, we tracked aAPC binding to cognate OT-II CD4^+^ T cells compared to non-cognate B6 CD4^+^ T cells. S1 aAPCs bound with significantly greater specificity to cognate CD4^+^ T cells across a range of doses (Fig. 2c, d).

The temperature of incubation as well as active cellular processes both affected the enrichment and recovery of diluted OT-II cells, as binding at 4 °C or metabolic inhibition with sodium azide (NaN_3_) each impaired the enrichment process (Supplementary Fig. 2a, b). To understand these findings, we tracked nanoparticle internalization over time by incubating cells with 200 nm particles conjugated with PE-labeled I-A^b^_OVA_ tetramers. Particles remaining on the surface of cells were subsequently detected with an anti-MHC II antibody. Interestingly, OT-II cells remained positive for the tetramer-labeled particles regardless of incubation time, temperature, or metabolic inhibition (Supplementary Fig. 2c); however, within the tetramer-positive cell populations, two hours of incubation at 37 °C in the absence of NaN_3_ led to a significant reduction in cells staining positive for MHC II, indicating internalization of the particles (Supplementary Fig. 2d, e). Loss of MHC II staining coincided with downregulation of TCRs, suggesting the particle internalization was TCR-mediated; indeed, non-cognate B6 CD4^+^ T cells that were incubated with particles in the same manner nonspecifically bound to but did not internalize aAPCs (Supplementary Fig. 2d, e). We confirmed aAPC internalization through confocal microscopy, observing a significant drop in the spatial correlation between particle and MHC II fluorescence at 37 °C in the absence of NaN_3_ (Supplementary Fig. 2f, g). To more closely assess the impact of aAPC internalization on the enrichment of rare CD4^+^ T cell populations, we performed an enrichment study as above with CFSE labeled OT-II CD4^+^ T cells, analyzing the surface binding versus internalization patterns of cognate CFSE^+^ OT-II and non-cognate CFSE^-^ B6 CD4^+^ T cells. We found that a two-hour incubation period at 37 °C in the absence of NaN_3_ allowed for the enrichment of OT-II cells with either surface-bound or internalized aAPCs (Supplementary Fig. 2h, i, left). In contrast, all other incubation conditions only enriched OT-II cells with surface-bound aAPCs. Moreover, enriched B6 cells from this incubation condition showed minimal particle internalization, despite nonspecifically binding to the aAPCs (Supplementary Fig. 2h, i, middle), demonstrating the antigen-specificity of internalization, even in mixed samples. Finally, unlike other conditions where OT-II CD4^+^ T cells were lost in the enrichment process even when bound at high levels with aAPCs, the majority of unenriched OT-II CD4^+^ T cells from samples incubated at 37 °C without metabolic inhibition were tetramer negative (Supplementary Fig. 2h, i, right), suggesting that aAPC interaction is more likely to lead to cell recovery in this condition.

To examine whether poor specific binding and enrichment of OT-II CD4^+^ T cells with S1/2 aAPCs was due to lower TCR-pMHC avidity compared to S1 aAPCs or non-specific CD28/αCD28 interactions, we examined the binding of lower density S1/I and S1/B aAPCs (Supplementary Fig. 1j) to cognate OT-II and non-cognate B6 CD4^+^ T cells. We found that while lower S1 density aAPCs bound less effectively to OT-II cells, their specific binding still remained significantly higher than their non-specific binding, unlike S1/2 aAPCs (Supplementary Fig. 3a, b). Indeed S1/I aAPCs yielded similar fold enrichment of diluted OT-II cells, as S1 aAPCs (Supplementary Fig. 3c), despite having a slightly lower density of I-A^b^ (Supplementary Fig. 1j). In contrast, 4.5 μm S1/2 aAPCs bound poorly to, and failed to enrich, cognate cells (Supplementary Fig. 3a–c).

The dose of aAPCs also affected the efficiency of enrichment and recovery of TCR transgenic OT-II and SMART-A1 lymphocytic choriomeningitis virus glycoprotein (I-A^b^ LCMV GP_61-80_) specific CD4^+^ T cells, with optimal cell enrichments and recoveries being achieved at 30 ng I-A^b^/10^6^ CD4^+^ T cells (Supplementary Fig. 3d–f). Using S1 + S2 aAPCs with this optimized enrichment protocol, we observed a 30–50-fold expansion of OT-II and SMART-A1 CD4^+^ T cells after 7 days (Fig. 2e, f). Likewise, the optimized protocol allowed us to expand a nearly 80% specific population of endogenous I-A^b^_OVA_ specific CD4^+^ T cells from a naïve B6 background in 7 days (Fig. 2g, h and Supplementary Fig. 3g, h). Based on estimated precursor frequencies of I-A^b^_OVA_ CD4^+^ T cells in B6 mice^24^, this corresponds to ~1000-fold expansion. In contrast, S1/2 aAPCs yielded a higher total number of CD4^+^ T cells on day 7, but both the percentage and number of antigen-specific CD4^+^ T cells were significantly reduced (Fig. 2g, h and Supplementary Fig. 3g, h), illustrating that separation of S1 and S2 can dramatically increase the frequency of rare antigen-specific CD4^+^ T cell populations.

### MHC II aAPCs promote CD4 + T cell cytotoxicity

**Fig. 3:**
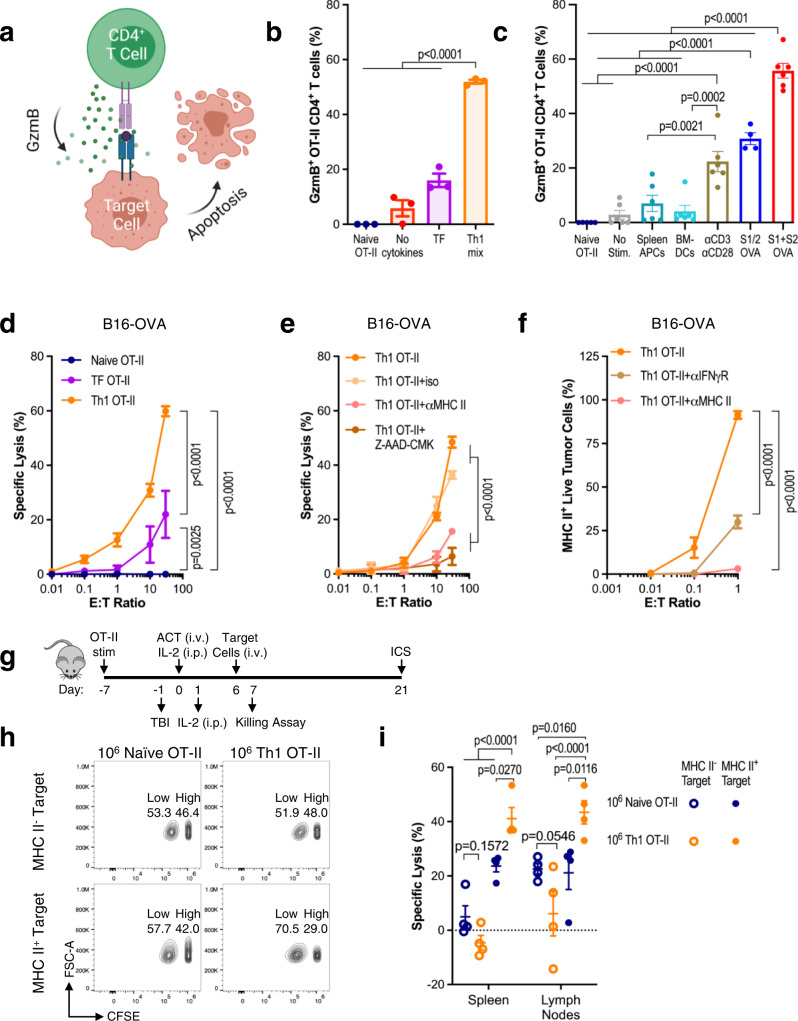
MHC II aAPCs promote CD4^+^ T cell cytotoxicity. **a** Schematic of direct CD4^+^ T cell lysis of target cells. Created with BioRender.com. **b** Granzyme B (GzmB) staining in OT-II cells stimulated with S1 + S2 aAPCs for 7 days in media containing: no cytokines, TF, or a Th1 mix. **c** Day 7 GzmB levels of OT-II cells stimulated in Th1 media with S1/2, S1 + S2, or αCD3/αCD28 aAPCs versus peptide-pulsed OT-II splenocytes or bone marrow-derived dendritic cells (BMDCs). **d** Specific lysis of B16-OVA tumor cells after overnight incubation with naïve or aAPC-stimulated OT-II cells (cultured in TF or Th1 media). Various effector-to-target (E:T) ratios are presented. **e** Specific lysis of B16-OVA cells after overnight incubation with aAPC-stimulated and Th1-skewed OT-II cells with MHC II antibody blocking or Z-AAD-CMK GzmB inhibition. **f** Percentage of MHC II-expressing live B16-OVA cells after overnight incubation with aAPC-stimulated Th1 OT-II cells and MHC II or IFN-γR antibody blocking. **g** Experimental overview of in vivo killing and cytokine production assays on naïve vs. aAPC-activated Th1 OT-II cells. **h**, **i** Specific lysis of OVA_323-339_ pulsed splenocytes six days after adoptive T cell transfer (ACT) of naïve or Th1 OT-II cells. Data in **b**–**f**, **i** represent mean ± standard error of the mean (s.e.m.) from three or more independent experiments. **b** *n* = 3 mice, **c** *n* = 4 (S1/2), 5 (Naïve), or 6 (No Stim., Spleen APCs, BMDCs, S1 + S2) mice, **d** *n* = 3 mice, **e** *n* = 3 (iso, MHC II, Z-AAD-CMK) or 4 (Th1 OT-II) mice, **f** *n* = 3 mice, **i** *n* = 4 mice/group, analyzed using a (**b**, **c**) one-way or (**d**–**f**, **i**) two-way ANOVA with Tukey’s multiple-comparisons test.

There have been published reports of CD4^+^ T cell acquisition of cytotoxic functions^12,25–27^ in various disease states, but there is no consistent method for producing them or studying them ex vivo. To assess the impact of MHC II aAPCs on CD4^+^ T cell cytotoxicity, we monitored the production of the serine protease Granzyme B (GzmB) and the associated lytic capacity of aAPC-activated CD4^+^ T cells (Fig. 3a). We found that induction of CD4^+^ T cell cytotoxicity was sensitive to both TCR engagement and the cytokine milieu. We observed a dramatic increase in GzmB levels when aAPC-activated CD4^+^ T cells were cultured in Th1 media compared to TF or cytokine-free media (Fig. 3b and Supplementary Fig. 4a). In Th1 media, S1 + S2 stimulation induced significantly higher levels of GzmB production than αCD3/αCD28 stimulation or the use of splenocytes or BMDCs pulsed with peptide (Fig. 3c and Supplementary Fig. 4b).

GzmB production increased over the course of S1 + S2 stimulation (Supplementary Fig. 4c) and was specifically dependent on the presence of IL-2 in the Th1 mix (Supplementary Fig. 4d, e). Additionally, we found that GzmB induction depended on soluble, as opposed to conjugated S2, and nano-, as opposed to microparticles, but did not depend on S1 density (Supplementary Fig. 4f). Consequently, S1 + S2 stimulated OT-II CD4^+^ T cells were able to lyse B16-OVA tumor cells in vitro when cultured in Th1 media (Fig. 3d and Supplementary Fig. 4g) in a GzmB and MHC II dependent manner (Fig. 3e). To elucidate how OT-II recognition of B16-OVA tumor cells was occurring, given that most tumors do not constitutively express MHC II, we monitored MHC II expression on live B16-OVA cells after coculture with S1 + S2 aAPC-activated OT-II cells, finding that the CD4^+^ T cells induce MHC II expression on B16-OVA in an IFN-γ dependent manner (Fig. 3f and Supplementary Fig. 4h). We next assessed the in vivo functional activity of S1 + S2 aAPC-activated OT-II CD4^+^ T cells by examining their lytic capacity and cytokine production 7 and 21 days post adoptive transfer into CD45.1 B6 mice (Fig. 3g). At day 7, aAPC-activated cells specifically lysed OVA_323-339_ pulsed target cells in an MHC II-restricted manner (Fig. 3h, i). Activated cells persisted through day 21, remaining T-bet positive (Supplementary Fig. 4i, j) and continuing to secrete IFN-γ and TNF-α (Supplementary Fig. 4k, l). Collectively, in vitro and in vivo cytotoxicity studies revealed that aAPCs can activate lytic programs in CD4^+^ T cells.

### MHC II aAPCs modulate CD4^+^ T cell helper function

**Fig. 4:**
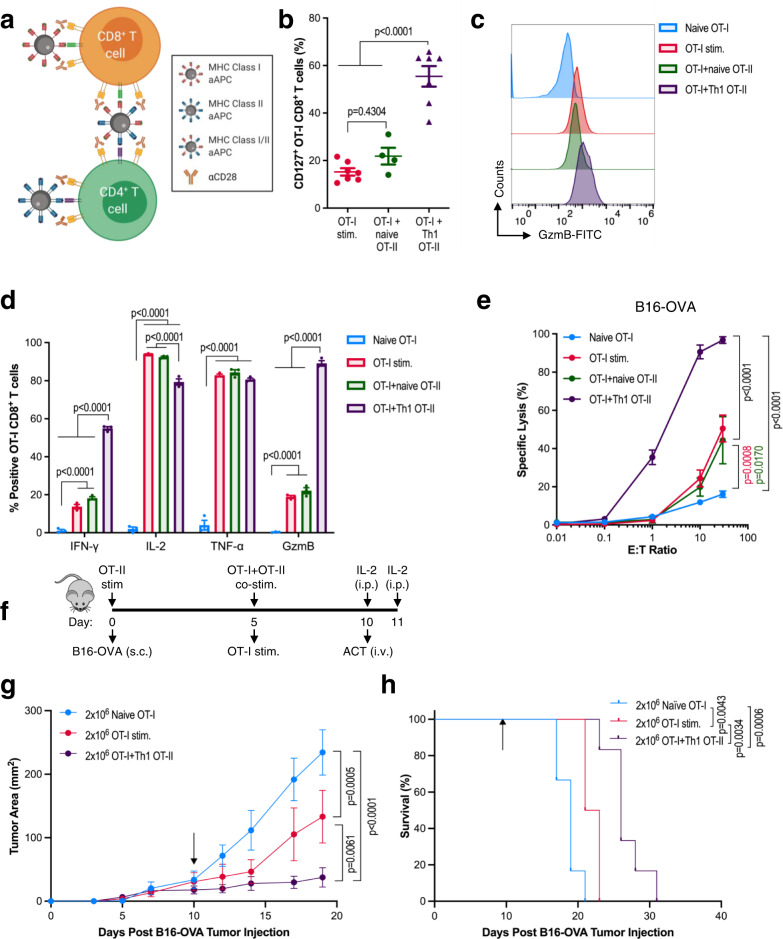
MHC II aAPCs modulate CD4^+^ T cell helper function. **a** Schematic showing separate (I + II) or joint presentation (I/II) of MHC I and MHC II on aAPCs to CD4^+^ and CD8^+^ T cells to facilitate cell-cell crosstalk. Created with BioRender.com. **b**–**h** OT-I CD8^+^ T cells were activated with MHC I K^b^_OVA_ aAPCs in TF-supplemented media either alone or in coculture with naïve or aAPC-stimulated Th1 OT-II cells and MHC II I-A^b^_OVA_ aAPCs. On day 7, **b** IL-7Rα (CD127) surface expression, **c** intracellular Granzyme B, **d** cytokine production, and **e** specific lysis of B16-OVA tumor cells after overnight incubation with CD8^+^ T cells were compared between stimulation cohorts. **f** Experimental overview of subcutaneous (s.c.) B16-OVA melanoma adoptive transfer model. **g** Tumor growth and **h** survival in mice subjected to adoptive transfer of OT-I CD8^+^ T cells that were either freshly harvested, activated in isolation, or co-activated with Th1 OT-II CD4^+^ T cells. The black arrows indicate the time of ACT. Data in **b**, **d**, **e** represent mean ± standard error of the mean (s.e.m.) or **g** mean ± standard deviation (s.d.) from three or more independent experiments. **b** *n* = 4 (OT-I + naïve OT-II) or 6 (OT-I stim, OT-I + Th1 OT-II) mice, **d** *n* = 3 mice, and **e** *n* = 3 (OT-I + naïve OT-II, OT-I + Th1 OT-II) or 4 (OT-I stim, Naïve OT-I) mice analyzed using a **b** one-way or **d**, **e** two-way ANOVA with Tukey’s multiple-comparisons test, **g**, **h** *n* = 6 mice/group analyzed using (**g**) a repeated measure two-way ANOVA with Tukey’s multiple-comparisons test or (**h**) log-rank test.

One objective in developing MHC II aAPCs was to produce a scalable approach for generating CD4^+^ T cells that could enhance the memory formation, function, and cytotoxicity of tumor-specific CD8^+^ T cells^13^. To do so, CD4^+^ and CD8^+^ T cells were co-activated either with separate MHC I and MHC II aAPCs (MHC I + II) or with a novel aAPC made by coupling nanoparticles with both MHC I and MHC II (MHC I/II) (Fig. 4a). In all cases, αCD28 was delivered in solution. We first assessed the impact of CD4^+^ and CD8^+^ co-activation on CD8^+^ T cell memory formation and function by co-culturing TCR transgenic K^b^ OVA_257-264_ specific OT-I CD8^+^ T cells (OT-I) at a 1:1 ratio with either naïve OT-II CD4^+^ T cells or OT-II CD4^+^ T cells activated with S1 + S2 aAPCs (Th1 OT-II). We found that co-stimulation of OT-I with Th1 OT-II cells using separate MHC I + II aAPCs led to an increase in effector memory CD8^+^ T cells that also expressed significantly higher levels of IL-7 receptor-alpha (IL-7Rα or CD127), a marker associated with long-lasting memory T cells (Fig. 4b and Supplementary Fig. 5a–c). The addition of Th1 OT-II cells also increased OT-I production of GzmB (Fig. 4c, d) and IFN-γ (Fig. 4d and Supplementary Fig. 5d). This effect was dependent on restimulation of the Th1 OT-II cells with either MHC I/II or MHC I + II aAPCs (Supplementary Fig. 5e–g). Furthermore, coculture with Th1 OT-II cells significantly boosted the in vitro cytotoxicity of TCR Transgenic OT-I, 2 C K^b^ SIY, and PMEL D^b^ gp100_25-33_ specific CD8^+^ T cells against B16-OVA (Fig. 4e and Supplementary Fig. 5h), B16-SIY (Supplementary Fig. 5i), and B16-F10 tumor cells (Supplementary Fig. 5j), respectively.

To determine whether CD4^+^ T cell help led to superior in vivo antitumor efficacy of CD8^+^ T cell therapies, we used an adoptive transfer model of preestablished murine melanoma (Fig. 4f). In this model, B6 mice were injected subcutaneously with B16-OVA tumor cells and then treated 10 days later with either naïve OT-I CD8^+^ T cells, aAPC-activated OT-I CD8^+^ T cells, or OT-I CD8^+^ T cells co-activated with Th1 OT-II CD4^+^ T cells using MHC I + II aAPCs. By the day of treatment, all of the cells in the coculture condition were CD8^+^, allowing direct comparisons of the antitumor function of CD8^+^ T cells across these three conditions (Supplementary Fig. 5k, l). We found that treatment with OT-I CD8^+^ T cells that had been cocultured with CD4^+^ T cells resulted in significantly improved B16-OVA antitumor control (Fig. 4g and Supplementary Fig. 5m) and enhanced survival (Fig. 4h) compared to both naïve or aAPC-activated OT-I CD8^+^ T cells. No tumors in treated mice fully regressed, potentially due to antigenic escape, a common limitation of this model^28^. Hence, both in vitro assays and in vivo disease models corroborated the beneficial role of aAPC-stimulated CD4^+^ T cells in boosting CD8^+^ T cell function.

### aAPC-mediated T cell help is driven by soluble factors and extends to endogenous CD8^+^ T cells

**Fig. 5:**
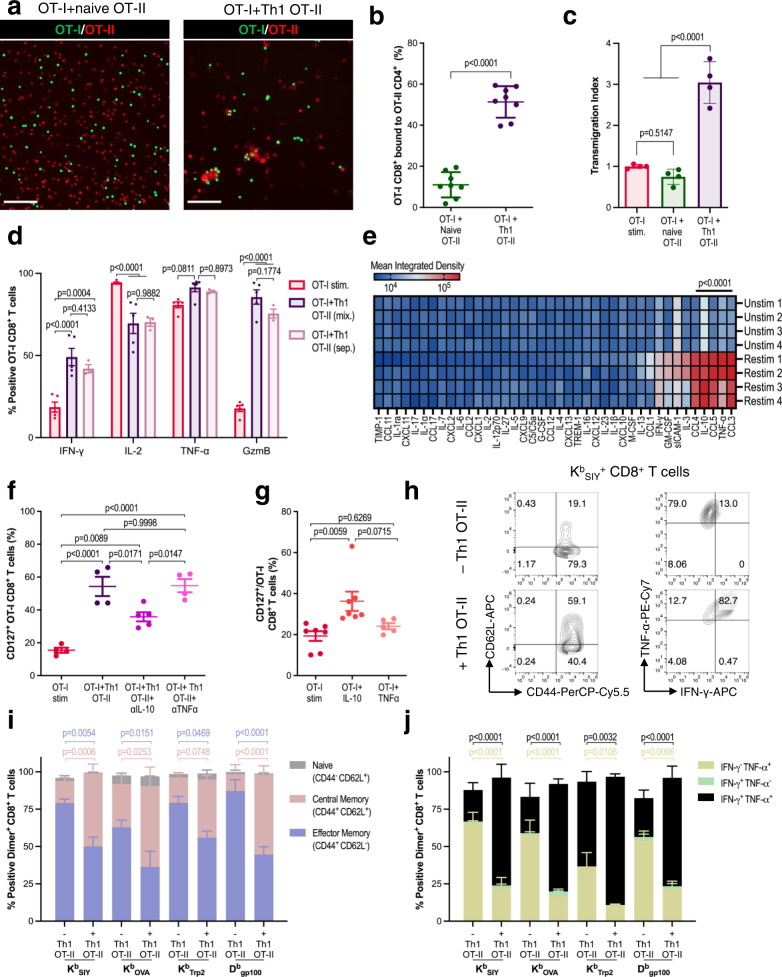
aAPC-mediated T cell help is driven by soluble factors and extends to endogenous CD8^+^ T cells. **a** Epifluorescent imaging and **b** co-localization analysis of OT-I cells (green) with naïve or Th1 OT-II cells (red) and MHC I/II aAPCs 24 h post-co-incubation. Scale bar: 100 μm. **c** Transmigration of OT-I cells towards naïve or Th1 OT-II cells relative to the basal medium. **d** Day 7 intracellular cytokine production of OT-I cells activated alone, separated (sep.) from, or mixed (mix.) with Th1 OT-II cells in a transwell plate. **e** Cytokine array heatmap depicting secreted proteins from unstimulated or re-stimulated Th1 OT-II cells. **f** CD127 expression of OT-I cells cocultured with Th1 OT-II cells with blocking antibodies targeting IL-10 and TNF-α. **g** CD127 expression of OT-I cells stimulated in IL-10 or TNF-α supplemented media. **h**–**j** K^b^_SIY_, K^b^_OVA_, K^b^_Trp2_, and D^b^_gp100_ specific CD8^+^ T cells were enriched from B6 mice and then expanded either alone or in coculture with Th1 OT-II cells. **h** Representative flow plots, **i** memory phenotype and **j** overall cytokine production of antigen-specific CD8^+^ T cells on day 7. Data in **b**–**d**, **f**, **g**, **i**, **j** represent mean ± standard error of the mean (s.e.m.) from three or more independent experiments. **b** *n* = 8, **c** *n* = 4 mice, **d** *n* = 3 (OT-I + Th1 OT-II sep.) or 5 (OT-I stim, OT-I + Th1 OT-II mix.) mice, **e** *n* = 4 mice, **f** *n* = 4 (OT-I + Th1 OT-II, OT-I + Th1 OT-II + αTNFα) or 5 (OT-I + Th1 OT-II + αIL-10) mice, **g** *n* = 5 (OT-I + TNFα) or 7 (OT-I stim, OT-I + IL-10) mice, and **i**, **j** *n* = 3 mice analyzed using an **b** unpaired Student’s *t*-test, two-tailed, **c**, **f**, **g** one-way, or **d**, **e**, **i**, **j** two-way ANOVA with Tukey’s multiple-comparisons test, mice.

To better understand the mechanisms underlying bolstered activity of CD8^+^ T cells cocultured with CD4^+^ T cells, we performed epifluorescent imaging of OT-I cells mixed with naïve or Th1 OT-II CD4^+^ T cells. After 24 h of co-incubation in the presence of MHC I/II aAPCs, OT-I CD8^+^ T cells had significantly more cell-cell interactions with Th1 OT-II than with naïve OT-II cells (Fig. 5a, b). Accordingly, Th1 OT-II cells induced significantly greater transmigration of OT-I than naïve OT-II cells (Fig. 5c). To assess whether this enhanced cell-cell interaction was complementary to or a requirement for improving OT-I function, we performed transwell assays, wherein OT-I and Th1 OT-II cells were either mixed together in the same well or separated by a 0.4-μm membrane. Interestingly, separation of OT-I and Th1 OT-II did not affect CD8^+^ memory skewing (Supplementary Fig. 6a) or function (Fig. 5d and Supplementary Fig. 6b, c), suggesting that MHC II aAPC-mediated CD4^+^ help occurred through soluble factors. Based on these results, we analyzed the supernatants of Th1 OT-II cells using a cytokine protein array (Fig. 5e and Supplementary Fig. 6d). The results indicated that the most highly abundant cytokines and chemokines were IL-10, TNF-α, CCL3, CCL4, and CCL5. Since chemokines CCL3, CCL4, and CCL5 primarily affect T cell migration, we focused on analyzing the impact of IL-10 and TNF-α. We found through blocking IL-10 and TNF-α in coculture experiments and adding exogenous IL-10 and TNF-α to OT-I only cultures, that IL-10 specifically impacts OT-I GzmB production (Supplementary Fig. 6e, f) and CD127 expression (Fig. 5f, g). Since help signals were observed to be delivered in solution, we next assessed how they would impact the memory phenotype and function of endogenous antigen-specific CD8^+^ T cells. To answer this question, we followed our existing protocol^23^ for enrichment and expansion of CD8^+^ T cells from naïve B6 mice, with or without Th1 OT-II CD4^+^ T cells added to the enriched fractions. We found that the addition of CD4^+^ T cells did not significantly alter the number of antigen-specific CD8^+^ T cells on day 7 (Supplementary Fig. 6g, h), but enhanced the central memory phenotype of antigen-specific CD8 + T cells (Fig. 5h, i), their IFN-γ production (Fig. 5h, j), and CD127 expression (Supplementary Fig. 6i).

### HLA II aAPCs stimulate functional antigen-specific human CD4^+^ T cells

**Fig. 6:**
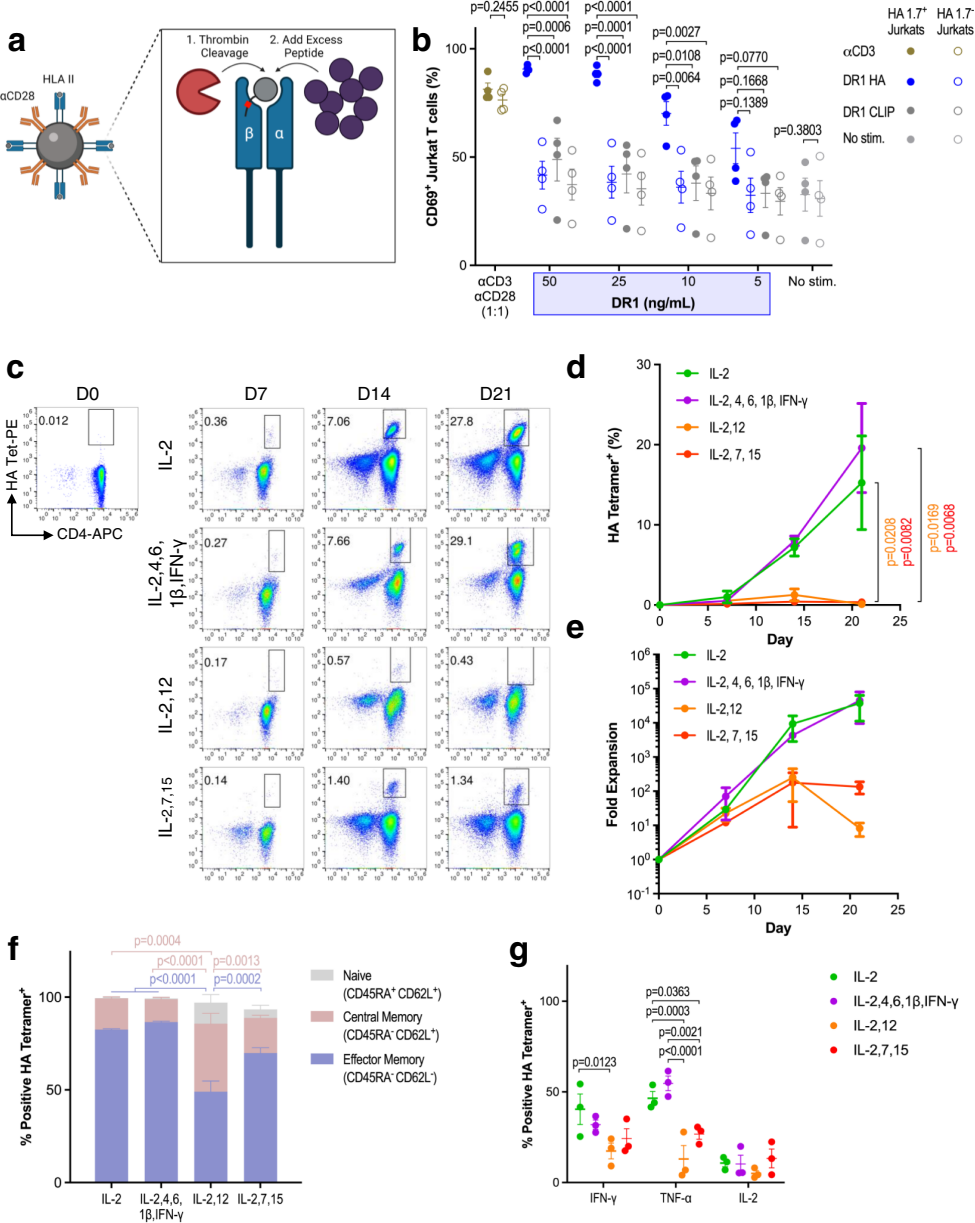
HLA II aAPCs stimulate functional antigen-specific human CD4^+^ T cells. **a** HLA II aAPC design includes HLA II molecules with cleavable thrombin linkers to facilitate peptide exchange. Created with BioRender.com. **b** CD69 induction on HA1.7 TCR-transfected Jurkat cells following stimulation with αCD3/αCD28 microparticles or titration of DR1/αCD28 aAPCs loaded with cognate hemagglutinin (DR1 HA) or non-cognate CLIP (DR1 CLIP) peptides. **c**–**g** Expansion of HA-specific CD4^+^ T cells from DRB1*04:01 healthy donor peripheral blood mononuclear cells (PBMC) treated with DR4 HA aAPCs in media supplemented with four different cytokine mixes: (i) IL-2; (ii) IL-2, IL-4, IL-6, IL-1β, and IFN-γ; (iii) IL-2 and IL-12; and (iv) IL-2, IL-7, and IL-15. **c** Representative tetramer staining, **d** frequency, and **e** fold expansion of DR4 HA CD4^+^ T cells on days 0, 7, 14, and 21. **f** Memory phenotype and **g** intracellular cytokine production of HA-specific CD4^+^ T cells on days 14 and 21. Data in **b**, **d**–**g** represent mean ± standard error of the mean (s.e.m.) from three or more independent experiments. **b** *n* = 4 and **d**–**g** *n* = 3 donors analyzed using a **d**, **e** repeated measure or **b**, **f**, **g** ordinary two-way ANOVA with Tukey’s multiple-comparisons test.

To establish whether the MHC II aAPC technology could be translated for human CD4^+^ T cell culture, we designed and expressed HLA class II monomers following a previously described system^29^ (Supplementary Fig. 7a, b). We then covalently attached these HLA molecules and αCD28 proteins to iron-dextran particles which could then be adapted to a range of target antigens through thrombin cleavage and peptide exchange (Fig. 6a). To assess the function and specificity of peptide-exchanged aAPCs, we exchanged HLA-DR1 aAPCs overnight with the hemagglutinin HA_306-318_ peptide and then monitored their ability to activate Jurkat cells transfected overnight with the HA_306-318_-recognizing HA1.7 TCR (Supplementary Fig. 7c). DR1 aAPCs loaded with the cognate HA peptide (DR1 HA) upregulated CD69, a T cell activation marker, specifically on the HA1.7 positive Jurkat cells; Moreover, unlike αCD3 based stimulation, which also activated HA1.7 negative Jurkat cells, DR1 HA aAPCs were specific for the HA1.7 expressing Jurkats (Fig. 6b and Supplementary Fig. 7d). We next assessed whether we could expand HA-specific CD4^+^ T cells from healthy DR4 donors, using DR4/αCD28 aAPCs. We compared expansion in four different cytokine mixes: IL-2 expansion media; IL-2, IL-4, IL-6, IL-1β, and IFN-γ human CD8^+^ culture media^21^; IL-2 and IL-12 Th1 skewing media; and IL-2, IL-7, and IL-15 memory skewing media. We found that both IL-2 media and IL-2,4,6,1β, and IFN-γ media resulted in a robust expansion of HA-specific CD4^+^ T cells from nearly undetectable precursor frequencies (Fig. 6c) to ~30% of the cell mixture (Fig. 6d), leading to nearly 100,000-fold expansion over the course of 21 days (Fig. 6e). Unlike with murine CD4^+^ T cells, this antigen-specific expansion was achieved without needing to separate S1 and S2. In contrast, IL-2 and 12 media and IL-2, 7, and 15 media only yielded modest expansions that declined after day 14. The resulting phenotype of the HA-specific CD4^+^ T cells from IL-2 or IL-2, 4, 6, 1β, and IFN-γ media was predominantly effector memory-like (Fig. 6f and Supplementary Fig. 7e) and ~30–40% of the cells were IFN-γ and TNF-α positive after antigen-specific restimulation (Fig. 6g and Supplementary Fig. 7f). Taken together, these results demonstrate that in the optimized cytokine milieu, HLA II aAPCs can expand rare antigen-specific human CD4^+^ T cells from endogenous repertoires.

## Discussion

Synthetic technologies for ex vivo expansion of T cells have continued to evolve over the past several decades to incorporate the breadth of biophysical and chemical cues that have been shown to affect T cell function^5^. These tools have thus far focused primarily on polyclonal T cell stimulation or expansion of antigen-specific CD8^+^ T cells^21,30–33^. However, for many disease or pathogen-specific applications, CD8^+^ T cells may play a less dominant role than other T cell subsets, particularly CD4^+^ T cells. Even in cancer, where CD8^+^ T cells are central to the therapeutic immune response, the antitumor function of these cells may be suboptimal without the addition of CD4^+^ T cell help at both the priming^34^ and effector^8^ stages. pMHC II-coated beads have been developed for in vivo induction of regulatory T cells in autoimmunity^35,36^. However, technologies that harness the effector or helper roles of CD4^+^ T cells have yet to be explored. To address these limitations, here we developed the MHC II aAPC, a nanoparticle platform for ex vivo expansion of antigen-specific murine and human CD4^+^ T cells. The platform confers several advantages over existing approaches to CD4^+^ T cell expansion, such as αCD3/αCD28 microparticles and peptide-pulsed autologous dendritic cells (DCs). αCD3/αCD28 microparticles provide non-specific stimulation that can result in the potential expansion of irrelevant or even pathogenic T cells^21,37^, presenting a hurdle for expansion of rare subsets of antigen-specific T cells. Autologous DCs provide antigen-specific stimulation; however, they require complex manufacturing steps, their availability is limited^38^, and the level and composition of signals they present to T cells are minimally controllable, which is of particular concern for cancer patients whose DCs are often dysfunctional^39,40^ or even immunosuppressive^41^. Here we showed that MHC II aAPCs could be used off the shelf to activate murine and human CD4^+^ T cells at levels similar to non-specific αCD3/αCD28 stimulation, while maintaining specificity for cognate CD4^+^ T cells. Furthermore, MHC II aAPCs were able to specifically expand initially undetectable antigen-specific murine and human CD4^+^ T cells from endogenous T cell repertoires. MHC II aAPCs could additionally be used in conjunction with existing synthetic platforms for ex vivo CD8^+^ T cell activation to relay crucial help signals from CD4^+^ T cells to a wide range of CD8^+^ T cells. These help signals, in turn, boosted memory formation, IFN-γ production, cytotoxicity, and in vivo antitumor control of antigen-specific CD8^+^ T cells. Thus, the MHC II aAPC presents a streamlined approach for the ex vivo generation of personalized CD4^+^ T cells and the provision of helper signals to CD8^+^ T cell therapies.

In addition to the clinical applications of the MHC II aAPC, it also provides a bottom-up approach for exploring CD4^+^ T cell biology. For instance, here, we show that MHC II aAPC stimulation results in the generation of cytotoxic CD4^+^ T cells, a phenotype which, thus far, has been observed primarily in vivo^12,25–27^. While confirming the importance of IL-2 in this process^42^, we also observed that differentiation of CD4^+^ T cells into cytotoxic T lymphocytes (CTL) occurred after stimulation with artificial and not endogenous APCs. Further comparisons of the signals presented by endogenous and artificial APCs may uncover the precise cues required for CD4^+^ CTL generation. Similarly, here we utilized the MHC II aAPC platform to study which CD4^+^ T cell cues directly enhance CD8^+^ T cell cytotoxicity and memory formation in the absence of confounding DC intermediaries. Interestingly, these studies revealed an immunostimulatory effect of IL-10 on CD8^+^ T cell cytotoxicity and effector function, in contrast with many studies that demonstrate IL-10 elicits T cell immunosuppression and anergy^43^. Our findings and other reported results^44,45^ indicate that the anti-inflammatory functions of IL-10 occur indirectly through suppression of APC function, whereas the direct effects of IL-10 on CD8^+^ T cells are stimulatory^46–49^. By providing a stable presentation of MHC and co-stimulatory molecules, aAPCs are uniquely poised to exploit the direct effects of IL-10 on enhancing CD8^+^ T cell antitumor function. In addition to the therapeutic implications of these findings, they are demonstrative of how a simplified approach using aAPCs can uncover additional aspects of the T cell help process that are difficult to study using traditional cellular approaches.

## Methods

### Mice

Permission for animal experiments was granted by the Johns Hopkins University’s Animal Care and Use Committee under Protocol Number: MO20M349. Similar numbers of male and female mice ranging from 8–12 weeks were used for experiments, and mice were maintained in adherence to committee guidelines. C57BL/6 (B6, Strain #: 000664), CD45.1 (Strain #: 002014), SMARTA-1 (Strain #: 030450), and OT-II (Strain #: 004194) mice were purchased from Jackson Laboratories (Bar Harbor, ME, USA). PMEL TCR transgenic mice (Jackson Strain #: 005023) were a gift from Nicholas Restifo (National Institutes of Health, MD, USA), and OT-IxRag2^−/−^ mice (Taconic, Strain #: 2334) were a gift from Jonathan Powell (Johns Hopkins University, MD, USA). 2 C TCR transgenic mice^50^ were maintained as heterozygotes by breeding on a B6 background. Mice were housed in a specific pathogen-free animal facility on a 12 light/12 dark light cycle, 65–75 °F, and 40–60% humidity. Experimental and control animals were co-housed.

### Human studies

All uses of human material in this study have been approved by the ethical committee of Johns Hopkins University, and all recruited volunteers provided written informed consent. Volunteers used in this study included two males, ages 27 and 55, and a female, age 32, each of whom was compensated for their blood donation ($20/80 mL). HLA DR4 typing was performed on donor PBMC using an NFLD.D.1 antibody^51^.

### Cells

B16-SIY was a gift from Thomas Gajewski (The University of Chicago, IL, USA), B16-F10 (ATCC no. CRL-6475) was a gift from Charles Drake (Johns Hopkins University, MD, USA), and B16-OVA was a gift from Jonathan Powell (Johns Hopkins University, MD, USA). Lymphoblastoid Cell Lines (LCL) were a gift from the Johns Hopkins Human Immunogenetics Laboratory (Johns Hopkins University, MD, USA). Human Jurkat T cells clone E6-1 (ATCC no. TIB-152) and Human Embryonic Kidney (HEK) 293 F cells (Thermo Invitrogen no. R79007) were a gift from Jamie Spangler (Johns Hopkins University, MD, USA). B16 cell lines were cultured in RPMI 1640 medium (Fisher Scientific) containing 10% FBS (Atlanta Biologicals) and 10 µM ciprofloxacin (Serologicals). B16-OVA and B16-SIY additionally received 400 μg/mL geneticin (Gibco). LCLs were cultured in RPMI 1640 medium containing 20% FBS, 200 mM L-glutamine (Gibco), 2 mM HEPES (Quality Biologicals), and 1X Pen/Strep (Gibco). Jurkat T cells were grown in RPMI 1640 media with 10% FBS and 100 U/ml penicillin-streptomycin (Sigma). Primary murine T cells were cultured in T cell media consisting of RPMI 1640 supplemented with l-glutamine, 1X non-essential amino acids (Gibco), 1 mM sodium pyruvate (Gibco), 0.4X MEM vitamin solution (Gibco), 92 µM 2-mercaptoethanol (Gibco), 10 µM ciprofloxacin, and 10% FBS—supplemented with a previously described T cell growth factor cocktail^18^, unless otherwise indicated. Primary human T cells were cultured in the described T cell culture media containing 10% AB serum (GeminiBio) instead of FBS and supplemented with additionally indicated cytokines. All cells and cell lines were maintained at 37 °C in a humidified atmosphere with 5% CO_2_.

### Reagents

Recombinant murine IL-2, IL-12p70, IFNγ, CCL3, CCL4, CCL5, IL-10, and TNFα and human IL-1β, IL-2, IL-4, IL-6, IL-7, IL-12, IL-15, and IFN-γ were purchased from Peprotech (Cranbury, NJ, USA). Recombinant human IL-2 used in adoptive cell transfer studies (Proleukin) was a gift from Prometheus Laboratories. I-A^b^ OVA_323-339_ (AAHAEINEA), I-A^b^ CLIP_87-101_ (PVSKMRMATPLLMQA), and I-A^b^ LCMV GP_66-77_ (DIYKGVYQFKSV) monomers and tetramers were provided by the NIH Tetramer Core Facility (Emory University, GA, USA). DR1 Plasmid was a gift from Luc Teyton (Scripps Research, CA, USA). Soluble DR1 and DR4 monomers were produced in-house, as described below^29^. Soluble Class I MHC‐Ig dimers were purified, biotinylated, and loaded with peptides according to previously described approaches^6^. The murine/human chimera HA1.7 T cell receptor was produced in-house, as described below. The HLA DR4-restricted NFLD.D.1 hybridoma supernatant was a gift from Sheila Drover (the Memorial University of Newfoundland, St. John’s, Canada)^51^. A list of all antibodies and their usage is summarized in Supplementary Table 1. Unlabeled murine and human monoclonal antibodies (anti-CD3 clones 145-2C11 and OKT-3, anti-CD28 clones 37.51 and 9.3, anti-OX40 clone OX-86, anti-IFNγR clone GR-20, anti-I-A/I-E clone M5/114, anti-TNFα clone XT3.11, and anti-IL-10 clone JES5-2A5) were purchased from BioXCell (West Lebanon, NH, USA). Fluorescently labeled monoclonal antibodies were purchased from BioLegend (San Diego, CA, USA), BD Biosciences (Franklin Lakes, NJ, USA), or eBioscience (San Diego, CA, USA), as indicated below, and used at a 1:100 dilution. OVA_323-339_ peptide was purchased from the Synthesis and Sequencing Facility (Johns Hopkins University, MD, USA). OVA_257-264_ (SIINFEKL), Trp2_180-188_ (SVYVFFDWL), SIY (SIYRYYGL), gp100_25-33_ (KVPRNQDWL), HA_306-318_ (PKYVKQNTLKLAT), and NY-ESO-1_157-170_ (SLLMWITQCFLPVF) peptides were purchased from Genscript (Piscataway, NJ, USA).

### Expression of human HLA-DR monomers

HLA-DR1 and DR4 monomers (Supplementary Table 2) were produced following a previously described approach^29^. Briefly, synthetic gene fragments (Twist Bioscience) for HLA-DR1 and DR4 α and β chains were separately cloned into the gWiz mammalian expression vector (Genlantis) using Gibson Assembly (New England Biolabs). The shared DRα chain vector consisted of the DRα gene (DRA*01:01) linked to a Fos leucine zipper dimerization domain that was further linked to a C-terminal hexahistidine tag. The distinct β chain vectors consisted of the Class II-associated invariant chain peptide (CLIP) followed by a thrombin cleavage site which was linked to the appropriate DRβ gene (DRB1*01:01 for HLA-DR1 or DRB1*04:01 for DR4). The DRβ gene was further linked to a Jun leucine zipper dimerization domain and C-terminal hexahistidine tag. Plasmids were purified using ZymoPURE II Plasmid Midiprep Kit (Zymo Research). All constructs were verified by Sanger sequencing.

HLA-DR1 and DR4 MHC proteins were expressed in a HEK 293-F mammalian cell expression system. HEK 293-F cells were cultivated in Freestyle 293 Expression Medium (Thermo Invitrogen), supplemented with 10 U/mL penicillin-streptomycin (Gibco). All cell lines were maintained at 37 °C in a humidified atmosphere with 5% CO_2_. HEK 293 F cells were maintained on a shaker set to 125 rpm.

HLA-DR1 and DR4 monomers were expressed recombinantly in the human embryonic kidney (HEK) 293-F cells via transient co-transfection of plasmids encoding the respective DRα and DRβ chains. DRα and DRβ chain plasmids were titrated in small-scale co-transfection tests to determine optimal DNA ratios for large-scale expression. HEK 293-F cells were grown to 1.2 × 10^6^ cells/mL and diluted to 1.0 × 10^6^ cells/mL on the day of transfection. Plasmid DNA (filter sterilized through a 0.22 µm PES filter [Corning]) and polyethyleneimine (PEI, Polysciences) were independently diluted to 0.05 and 0.1 mg/mL, respectively, in OptiPro medium (Thermo Invitrogen), and incubated at 20 °C for 15 min. Equal volumes of diluted DNA and PEI were mixed and incubated at 20 °C for an additional 15 min. Subsequently, the DNA/PEI mixture (40 mL per Liter cells) was added to a flask containing the diluted HEK cells, which was then incubated at 37 °C with shaking for 3–5 days. Secreted protein was harvested from HEK 293-F cell supernatants via Ni-NTA (Expedeon) affinity chromatography, followed by size-exclusion chromatography on an ÄKTA fast protein liquid chromatography (FPLC) instrument using a Superdex 200 column (Cytiva). All proteins were stored in HEPES-buffered saline (HBS, 150 mM NaCl in 10 mM HEPES pH 7.3). Purity was verified by SDS-PAGE analysis.

### Biotinylation, thrombin cleavage, peptide exchange, and tetramerization of human HLA-DR monomers

For the preparation of biotinylated HLA-DR1 and DR4, a C-terminal biotin acceptor peptide (BAP) GLNDIFEAQKIEWHE sequence was added to the previously described HLA-DR expression vectors. Following transfection and Ni-NTA affinity chromatography, the HLA-DR monomers were biotinylated with the soluble BirA ligase enzyme in 0.5 mM Bicine pH 8.3, 100 mM ATP, 100 mM magnesium acetate, and 500 mM biotin (Sigma). After overnight incubation at 4 °C, excess biotin was removed by size-exclusion chromatography on an ÄKTA FPLC instrument using a Superdex 200 column (Cytiva). To confirm the covalent attachment of biotin, at least 1 μg of each biotinylated HLA-DR protein was incubated with 2 mL of streptavidin (5 mg/mL, MilliporeSigma) at 20 °C for 5 min followed by SDS-PAGE analysis to confirm a shift in molecular weight.

CLIP peptides were cleaved by incubating DR proteins with 20 U of thrombin (Novagen, Madison, WI) per milligram of monomer at 37 °C for 2 h. Peptide exchange was then performed by adjusting the concentration of monomer to 3.3 μM in a peptide exchange buffer consisting of 50 mM sodium citrate pH 5.2, 1% octylglucoside (Thermo Fisher), 100 mM NaCl and 1X protease inhibitor cocktail (Roche) and incubating with 50 μM of peptide overnight at 37 °C. To remove excess peptides, monomers were then washed three times in PBS with a 10 kDa MWCO concentrator (Sigma) and then frozen in small aliquots at −80 °C.

Multimerization reactions were performed through incremental addition of fluorescent streptavidin molecules (Agilent) to biotinylated monomer at 20 °C to reach a final streptavidin to monomer ratio of 1:3.5.

### Synthesis of aAPCs

Murine I-A^b^ CLIP and I-A^b^ OVA and murine and human αCD3/αCD28 microparticles (Dynal, Lake Success, New York) were synthesized according to the manufacturer’s instructions and as previously described in ref. 6. Murine and human nanoparticle aAPCs were synthesized as previously described in ref. 23 and in accordance with the manufacturer’s instructions by incubating 200 nm NHS-activated magnetic beads (Ocean Nanotech, Springdale, AR, USA) with either I-A^b^, DR1, DR4, K^b^-Ig, or D^b^-Ig monomers, dimers, or fluorescently labeled tetramers. Combined Signal 1 and Signal 2, Signal 1 and Isotype, or Signal 1 and BSA aAPCs were produced by premixing monomers or dimers at a 1:1 or 1:3 molar ratio, as indicated, with a mouse or human αCD28, isotype Armenian hamster IgG antibodies Clone HTK888 (Biolegend), or Bovine Serum Albumin (GeminiBio). Combined MHC I and MHC II aAPCs were produced by premixing I-A^b^ monomers with K^b^-Ig dimers at a 1:1 molar ratio.

Human aAPCs underwent thrombin cleavage and peptide exchange post conjugation of DR-CLIP proteins. Briefly, aAPCs were incubated with 40 units of thrombin per milligram of conjugated DR protein at 37 °C for 2 h. Particles were then magnetically washed and resuspended at 30 nM conjugated protein in peptide exchange buffer and then incubated overnight at 37 °C with 3 μM peptide. Finally, particles were washed and resuspended either in storage buffer (1X PBS and 0.05% BSA) or human T cell culture media.

### Characterization of aAPCs

Nanoparticles were sized using a Zetasiser DLS and imaged using Transmission Electron Microscopy (TEM). For TEM, iron-dextran nanoparticles were allowed to adhere on carbon-coated copper support grids (EMS CF400-Cu-UL) for 2 min, rinsed three times with deionized water, and allowed to dry at 20 °C. The grids were mounted and imaged on a transmission electron microscope (Hitachi 7600) at an acceleration voltage of 80 kV.

Protein conjugation to Dynal microparticles was characterized by staining microparticles with FITC labeled secondary antibodies and then comparing them to a standard curve based on a Quantum FITC-5 MESF fluorescence quantification kit (Bangs Laboratories). Protein conjugation to nanoparticle aAPCs was performed as previously described in ref. 52 by staining particles with FITC labeled secondary antibodies, magnetically washing the particles, and then comparing their absorbance at 405 nm (Beckman Coulter AD340) and fluorescence at 485 nm (Fisher Scientific Varioskan LUX) to standard curves of the known bead and protein concentrations, respectively. The following secondary antibodies were used: FITC anti-hamster IgG clone G94-56 (BD Biosciences) for murine αCD3, FITC anti-hamster IgG clone G192-1 (BD Biosciences) for murine αCD28, FITC anti-mouse I-A/I-E clone M5/114.15.2 (BioLegend) for murine I-A^b^, FITC anti-mouse Ig λ1 λ2 λ3 light chain clone R26-46 (BD Biosciences) for murine K^b^-Ig and D^b^-Ig, FITC anti-mouse IgG2a clone R19-15 (BD Biosciences) for human αCD3 and αCD28, and FITC anti-human HLA-DR clone L243 (BioLegend) for human DR1 and DR4. For fluorescent tetramer-labeled nanoparticles, the protein concentration per nanoparticle was determined by comparing the fluorescence of the particles to a standard curve of the unconjugated fluorescent tetramer.

### T cell isolation

OT-II, SMART-A1, or B6 mice were used for CD4^+^ expansions, and OT-I, 2 C, PMEL, and B6 mice were used for CD8^+^ expansions. Spleens and lymph nodes were harvested from 8 to 12-week-old mice and processed through a 70-μm cell strainer. Then, CD4^+^ and CD8^+^ T cells were isolated using corresponding no-touch isolation kits and magnetic columns from Miltenyi Biotech (Auburn, CA, USA) according to the manufacturer’s instructions.

For human isolations, blood was drawn from healthy donors per JHU IRB-approved protocols and PBMC were isolated by Ficoll-Paque PLUS (GE Healthcare) density gradient centrifugation. Cells were cryopreserved in a 90% FBS, 10% DMSO solution at 10^7^ cells/mL and stored in liquid nitrogen. Prior to use, cryopreserved PBMC were thawed with 50 U/mL benzonase Nuclease HC (EMD Millipore), washed, and then incubated overnight in a T cell culture medium at 37 °C. The following morning, CD4^+^ T cells were purified using no-touch CD4^+^ isolation kits and magnetic columns (Miltenyi).

### Bone marrow-derived dendritic cell isolation

Bone marrow-derived dendritic cells (BMDC) were generated following a well-established approach^53^. Marrow was flushed from femurs and tibia of B6 mice, filtered, red blood cells lysed, washed, and cultured in nontreated six-well plates at 1 × 10^6^ cells/mL in DC media containing RPMI 1640 media (Gibco) supplemented with 10% FBS, 1% Pen/Strep (Gibco), 50 μM 2-mercaptoethanol (Gibco), and 20 ng/mL GM-CSF (Peprotech). On day 3, cells were refed with DC media containing 40 ng/mL GM-CSF. On day 6, 50% of cell supernatant was replaced with DC media containing 20 ng/mL GM-CSF. On day 8, non-adherent or loosely adherent cells were harvested and matured overnight by replating cells at 1 × 10^6^ cells/mL in DC media containing 100 ng/mL lipopolysaccharide (Sigma Aldrich), 20 ng/mL GM-CSF, and 1 μM of peptide. Prior to stimulation of CD4^+^ T cells, DC maturation was confirmed via flow cytometry by staining for FITC anti-mouse CD11b clone M1/70 (BD Biosciences), PerCP-Cy5.5 anti-mouse CD11c clone N418 (BioLegend), APC anti-mouse CD86 clone GL-1 (BioLegend), Live/Dead Fixable Violet (Invitrogen), BV605 anti-mouse F4/80 clone BM8 (BioLegend), PE anti-mouse CD80 clone 16-10A1 (BioLegend), and PE-Cy7 anti-mouse I-A/I-E clone M5/114.15.2 (BioLegend).

### Ex vivo T cell expansion

Isolated murine CD4^+^ T cells were cultured in T cell culture media with the addition of either a previously described optimized T cell growth factor cocktail (TF)^18^, IL-2 (10 ng/mL), or various combinations of a Th1 skewing media composed of IL-2, IL-12p70, and IFN-γ (each at 10 ng/mL). Cells were plated on day 0 at 10^5^ cells/mL and refed on day 3 of culture, with half of the initial volume of T cell culture media and twice the concentration of cytokines. On day 0, micro-aAPCs were added at a 1:1 particle-to-cell ratio, whereas nano-aAPCs were added at a concentration of 80 ng/mL of conjugated I-A^b^, unless otherwise indicated. For aAPCs lacking Signal 2 on their surface, soluble αCD28 was added at a concentration of 1 μg/mL unless otherwise indicated. For peptide-based stimulations, isolated splenocytes were plated at 8 × 10^5^ cells/mL in T cell culture media with the addition of 1 μg/mL of peptide. For BMDC-based stimulations, murine CD4^+^ T cells were plated at 10^5^ cells/mL and at a 1:1 ratio with mature BMDCs in T cell culture media.

Murine CD4^+^ T cell proliferation was assessed by labeling a subset of isolated CD4^+^ T cells on day 0 with carboxyfluorescein succinimidyl ester (CFSE, Invitrogen). Cells were incubated with 5 μM dye in T cell culture media at 37 °C for 20 min, washed and plated as above, and on day 3 of culture, harvested and assessed for CFSE dilutions on a BD FACSCalibur flow cytometer. Another subset of unlabeled cells was plated as above and, on day 7, harvested, stained with Trypan blue to exclude dead cells, and then manually counted with a hemocytometer. Fold expansion was calculated as the ratio of live cells on days 7 and 0. Cell phenotype and function was assessed, as described below.

Isolated murine CD8^+^ T cells were cultured as above in T cell culture media supplemented with TF. Class I aAPCs were added at a concentration of 30 ng/mL of conjugated K^b^ or D^b^ and 1 μg/mL soluble αCD28 unless otherwise indicated. Cells were refed as above on day 3 and then harvested and counted on day 7 for functional and phenotypic analyses. For some experiments, T cell culture media was additionally supplemented at day 0 with 25 ng/mL IL-10 or 5 ng/mL TNF-α, and then refed with double these concentrations and half the initial volume on day 3.

For murine CD4^+^ and CD8^+^ coculture experiments, CD8^+^ T cells were mixed at a 1:1 ratio with either freshly isolated CD4^+^ T cells or CD4^+^ T cells activated with S1 + S2 aAPCs (80 ng/mL conjugated I-A^b^ and 1 μg/mL αCD28) for 5 days in Th1 media. The CD4:CD8 mixture was then plated at 10^5^ cells/mL in T cell culture media supplemented with TF, MHC I aAPCs (30 ng/mL), MHC II aAPCs (80 ng/mL), and soluble αCD28 (1 μg/mL), unless otherwise indicated. For some experiments, T cell culture media was additionally supplemented on days 0 and 3 with 1 μg/mL IL-10 or TNF-α blocking antibodies. Cells were refed as above on day 3 and harvested and counted on day 7 for further functional and phenotypic analyses. The relative ratios of CD4^+^ and CD8^+^ T cells over the coculture period was tracked via flow cytometry by staining cells with APC anti-mouse CD4 clone GK1.5 (Biolegend), PE anti-mouse CD3 clone 17A2 (Biolegend), FITC anti-mouse CD8a clone 53-6.7 (BD Biosciences), and Live/Dead Fixable Violet (Invitrogen). CD4^+^ and CD8^+^ coculture experiments were also performed in 0.4-μm pore-size polycarbonate membranes transwell plates (Costar). 10^5^ OT-I CD8^+^ T cells were placed in the lower compartment in 0.75 mL of T cell culture media supplemented with T cell growth factor, MHC I aAPCs (30 ng/mL conjugated K^b^), and 1 μg/mL αCD28. 10^5^ Day 5 Th1 OT-II CD4^+^ T cells were either separated in the upper or mixed with the CD8^+^ T cells in the lower compartment in an additional 0.75 mL of T cell culture media supplemented with TF, MHC II aAPCs (80 ng/mL conjugated I-A^b^), and 1 μg/mL αCD28. Cells were refed as above on day 3 and harvested and counted on day 7 for further functional and phenotypic analyses.

For human T cell expansions, the day 0 precursor frequencies of HA_306-318_ CD4^+^ T cells was assessed through tetramer staining. Isolated CD4^+^ T cells were then seeded at 10^6^ cells/mL in human T cell culture medium with indicated cytokines, and peptide-exchanged Class II aAPCs were added at a concentration of 30 ng/mL of conjugated DR4. On days 3, 5, 10, 12, 17, and 19, cells were refed with one-quarter of the initial volume of T cell culture media and twice the concentration of cytokines, and on days 7, 14, and 21, cells were harvested, counted, and assessed for antigen-specificity, phenotype, and function. On days 7 and 14, cells were additionally replated with fresh media, cytokines, and aAPCs at 5 × 10^5^ cells/mL and 100 ng/mL DR4 (day 7) and 3 × 10^5^ cells/mL and 100 ng/mL DR4 (day 14), respectively. Fold proliferation on days 7, 14, and 21 was calculated as the ratio of live tetramer-positive CD4^+^ T cells (total number of cells multiplied by the percentage of live lymphocytes that were both CD4 and tetramer-positive) at the current and previous time points. Representative gating strategies for ex vivo T cell expansion studies can be found in Supplementary Fig. 8a.

### Ex vivo T cell phenotypic studies

Lineage-specific transcription factors of naïve or expanded murine CD4^+^ T cells were analyzed by washing cells and staining them for 15 min at 4 °C with Live/Dead Fixable Aqua (Invitrogen) and APC-Cyanine7 anti-mouse CD4 clone GK1.5 (BioLegend). Cells were then washed, fixed, and permeabilized using the Foxp3 Transcription Factor Staining Buffer Set (eBioscience), and then stained for FITC anti-mouse Foxp3 clone FJK-16s (eBioscience), PerCp-Cyanine5.5 anti-mouse/human T-bet clone eBio4B10 (eBioscience), APC anti-mouse/human RORγT clone AFKJS-9 (eBioscience), and PE/Cyanine7 anti-mouse/human Gata3 clone TWAJ (eBioscience), or their corresponding isotypes. Finally, cells were washed and resuspended in FACS wash buffer (1X PBS, 2% FBS, 0.5% sodium azide) and then analyzed on an Attune NxT Flow Cytometer.

The memory phenotype of naïve or expanded murine CD4^+^ or CD8^+^ T cells was analyzed by harvesting cells and then washing and staining them for 15 min at 4 °C with Live/Dead Fixable Violet (Invitrogen), PE anti-mouse CD3 clone 17A2 (BioLegend), APC/Cyanine7 anti-mouse CD4 clone GK1.5 (BioLegend) or APC/Cyanine7 anti-mouse CD8a clone 53-6.7 (BioLegend), Alexa Fluor 488 anti-mouse CD127 clone A7R34 (BioLegend), PerCP-Cy5.5 anti-mouse CD44 clone IM7 (BioLegend), APC anti-mouse CD62L clone MEL-14 (BioLegend), Brilliant Violet 605 anti-mouse/human KLRG1 clone 2F1/KLRG1 (BioLegend), and PE/Cyanine7 anti-mouse CD197 (CCR7) clone 4B12 (BioLegend), or their corresponding isotypes. For rare T cell analysis, PE-labeled multimer staining was substituted for anti-CD3 (see below) and performed prior to other surface marker staining.

The memory phenotype of human CD4^+^ T cells was analyzed by first staining cells with PE-labeled tetramers (see below), and then staining them for 15 min at 4 °C with Live/Dead Fixable Aqua, PE/Cyanine7 anti-human CD4 clone A161A1 (BioLegend), FITC anti-human CD45RA clone HI100 (BioLegend), APC/Cyanine7 anti-human CD62L clone DREG-56 (BioLegend), PerCP-Cyanine5.5 anti-human CD69 clone FN50 (BioLegend), APC anti-human CD103 clone Ber-ACT8 (BioLegend), and Brilliant Violet 421 anti-human CD122 clone TU27 (BioLegend), or their corresponding isotypes. Representative gating strategies for ex vivo T cell phenotypic studies can be found in Supplementary Fig. 8a.

### Ex vivo T cell functional studies

Intracellular cytokine staining of murine CD4^+^ and CD8^+^ T cells was performed by diluting them to ~2 × 10^6^ cells/mL in T cell culture media and incubating them at 37 °C for 6 h with 1X cytokine activation cocktail (BioLegend) and GolgiPlug (BD Biosciences). No stimulation controls were received, only GolgiPlug. Following incubation, cells were washed and stained with PerCP anti-mouse CD4 clone RM4-5 (BioLegend) or PerCP anti-mouse CD8 clone 53-6.7 (Biolegend) and Live/Dead Fixable Aqua (Invitrogen) for 15 min at 4 °C. Cells were then fixed and permeabilized overnight with the Cytofix/Cytoperm Fixation/Permeabilization kit (BD Biosciences), washed, and stained with APC anti-mouse IFN-γ clone XMG1.2 (BioLegend), PE/Cyanine7 anti-mouse TNF-α clone MP6-XT22 (BioLegend), PE anti-mouse IL-2 clone JES6-5H4 (BioLegend), and FITC anti-mouse/human Granzyme B clone GB11 (BioLegend). Cells were then washed and resuspended in FACS wash buffer and analyzed on an Attune NxT Flow Cytometer.

For cytokine analysis of antigen-specific murine CD8^+^ T cells, a similar assay was used with the following modifications. Prior to stimulation, T cells were stained with cognate and non-cognate biotinylated pMHC-Ig dimers (see below), washed, and then re-stimulated. After the 6 h incubation, cells were washed and stained with PerCP anti-mouse CD8 clone 53-6.7 (BioLegend), PE-labeled streptavidin (BD Biosciences), and Live/Dead Fixable Aqua (Invitrogen) for 15 min at 4 °C. Cells were then fixed and permeabilized and stained with APC anti-mouse IFN-γ clone XMG1.2 (BioLegend), PE/Cyanine7 anti-mouse TNF-α clone MP6-XT22 (BioLegend), and FITC anti-mouse/human Granzyme B clone GB11 (BioLegend). Cells were then washed and resuspended in FACS wash buffer and analyzed on an Attune NxT Flow Cytometer.

Antigen-specific human CD4^+^ T cell cytokine analysis was performed by pulsing LCLs with 10 μg/mL cognate (HA_306-318_) or irrelevant (NY-ESO-1_161-180_) peptide for 1 h at 20 °C, washing, and then incubating them 1:1 with T cells in human T cell culture media containing GolgiPlug for 5 h at 37 °C. Tetramer staining was begun 50 minutes prior to the end of the 5 h incubation (see below). Afterwards, cells were washed and stained for APC anti-human CD4 clone OKT4 (BioLegend) and Live/Dead Fixable Aqua (Invitrogen). Cells were then fixed and permeabilized as above and stained for FITC anti-human IFN-γ clone 4 S.B3 (BioLegend), PerCP-Cy5.5 anti-human IL-2 clone MQ1-17H12 (BioLegend), Pacific Blue anti-mouse/human Granzyme B clone GB11 (BioLegend), and PE/Cyanine7 anti-human TNF-α clone MAb11 (BioLegend). Cells were then washed and resuspended in FACS wash buffer and analyzed on an Attune NxT Flow Cytometer. Representative gating strategies for ex vivo T cell functional studies can be found in Supplementary Fig. 8a.

In vitro killing assays of murine CD4^+^ and CD8^+^ T cells were performed as previously described in ref. 21 by labeling 5 × 10^6^ B16 tumor cells with 5 μM CFSE dye (Invitrogen) at 37 °C for 20 min in 1 mL PBS. The reaction was quenched by adding 5 mL FBS and incubating cells at 37 °C for 5 min. Tumor cells were plated at 5 × 10^4^ cells/mL in ultra-low cluster 96-well plates (Costar) co-incubated with T cells at varying effector-to target ratios (30:1, 10:1, 1:1, 0.1:1, 0.01:1, and 0:1) at 37 °C for 16 h. For blocking studies, anti-I-A/I-E clone M5/114 (BioXcell) or anti-IFNγR clone GR-20 (BioXcell), as well as their corresponding isotype controls, were added at 10 μg/mL, while Granzyme B inhibitor Z-AAD-CMK (Calbiochem) was added at 25 μM. Cells were then treated with trypsin to detach plate-bound tumor cells, stained for 15 min at 4 °C with Live/Dead Fixable Aqua (Invitrogen) and APC anti-mouse CD4 clone GK1.5 (BioLegend) or APC anti-mouse CD8a clone 53-6.7 (BioLegend), washed, and then run analyzed on an Attune NxT Flow Cytometer. To monitor MHC II expression on live tumor cells, cells were instead stained with Live/Dead Fixable Violet (Invitrogen), APC anti-mouse CD4 clone GK1.5 (BioLegend), and PE/Cyanine7 anti-mouse I-A/I-E clone M5/114.15.2 (BioLegend). Representative gating strategies for in vitro killing assays can be found in Supplementary Fig. 8b.

### Multimer staining

Murine CD4^+^ T cell tetramer staining was performed by incubating 1 × 10^5^ cells at 37 °C for 2 h with 60 μg/mL cognate and non-cognate I-A^b^ tetramers (NIH Tetramer Core Facility) in a T cell culture medium. Cells were then washed in PBS, stained with APC anti-mouse CD4 clone GK1.5 (BioLegend) and Live/Dead Fixable Green (Invitrogen) for 15 min at 4 °C, washed and resuspended in FACS Wash Buffer, and then analyzed on an Attune NxT Flow Cytometer.

Murine CD8^+^ T cell dimer staining was performed by incubating 1 × 10^5^ cells at 4 °C for 1 h with 10 μg/mL cognate and non-cognate biotinylated K^b^-Ig or D^b^-Ig dimers (in-house) in FACS Wash Buffer. Cells were then washed in PBS, stained with APC anti-mouse CD8a clone 53-6.7 (BioLegend) and Live/Dead Fixable Green (Invitrogen) for 15 min at 4 °C, washed and resuspended in FACS Wash Buffer, and then analyzed on an Attune NxT Flow Cytometer.

Human CD4^+^ T cell tetramer staining was performed by incubating 1 × 10^5^ cells at 20 °C for 5 min with 40 μL/mL Human TruStain FcX Fc Receptor Blocking Solution (BioLegend) in a T cell culture medium. An additional 20-min incubation at 37 °C with 50 nM dasatinib (Axon Medchem) followed by a 30 min incubation at 37 °C with 20 μg/mL cognate and non-cognate tetramers (in-house) was then done. Cells were then washed in PBS, stained with APC anti-human CD4 clone OKT4 (BioLegend) and Life/Dead Fixable Green (Invitrogen) for 15 min at 4 °C, washed and resuspended in FACS Wash Buffer, and then analyzed on an Attune NxT Flow Cytometer. Representative gating strategies for multimer staining can be found in Supplementary Fig. 8a.

### T cell binding, internalization, enrichment, and combined enrichment and expansion

Murine CD4^+^ T cell binding studies were performed by incubating 1 × 10^5^ recently isolated OT-II, SMART-A1, or B6 CD4^+^ T cells for 30 min at 37 °C in T cell culture media with varying concentrations of nano- and micro-aAPCs. Cells were then washed and stained for 15 min at 4 °C in FACS Wash Buffer with FITC anti-mouse I-A/I-E clone M5/114.15.2 (BioLegend) and APC anti-mouse CD4 clone GK1.5 (BioLegend) to detect aAPC-bound CD4^+^ T cells, washed again, and then analyzed on a BD FACSCalibur Flow Cytometer.

Murine CD4^+^ T cell internalization studies were performed as above using nanoparticles coated with PE-labeled I-A^b^_OVA_ tetramers at 80 ng I-A^b^/10^5^ CD4^+^ T cells. The incubation time was varied between 30 and 120 min, incubation temperature between 4 °C and 37 °C, and incubation media between T cell culture with and without 0.5% sodium azide (NaN_3_) supplementation. Cells were then washed and stained for 15 min at 4 °C in FACS Wash Buffer with FITC anti-mouse TCR β chain clone H57-597 (BioLegend), APC anti-mouse CD4 clone GK1.5 (BioLegend), and PE-Cy7 anti-mouse I-A/I-E clone M5/114.15.2 (BioLegend). Samples were then washed again and analyzed on an Attune NxT flow cytometer for the percentage of cells with surface-bound (Tetramer^+^MHC II^+^) versus internalized (Tetramer^+^MHC II^−^) aAPCs.

OT-II doped enrichment studies were performed by CFSE labeling recently isolated OT-II CD4^+^ T cells with 5 μM CFSE (Invitrogen) in T cell culture medium for 20 min at 37 °C and then diluting them 1:1000 with recently isolated, unlabeled B6 CD4^+^ T cells. Cells were then incubated for 2 h with micro- or nano-aAPCs at 37 °C in T cell culture media and then magnetically enriched using a 96-well ring magnet^23^. For some experiments, the incubation was performed at 4 °C or with T cell culture media supplemented with 0.5% sodium azide (NaN_3_). The enriched fraction was then counted with a hemocytometer, washed, and stained at 4 °C for 15 min with APC anti-mouse CD4 clone GK1.5 (BioLegend) in FACS Wash Buffer. Cells were then washed and analyzed on a BD FACSCalibur Flow Cytometer. Fold enrichment and percent cell recovery were calculated by taking the ratio of both the frequency and number of CFSE + CD4^+^ T cells pre and post-enrichment. To track aAPC internalization during the enrichment process, diluted cells were incubated with nano-aAPCs conjugated with PE-labeled tetramers at 30 ng I-A^b^/10^6^ CD4^+^ T cells, as above. Both the enriched and unenriched fractions were collected, counted with a hemocytometer, washed, and stained at 4 °C for 15 min with PerCP anti-mouse CD4 clone RM4-5 (Biolegend), PE-Cy7 anti-mouse I-A/I-E clone M5/114.15.2 (BioLegend), and Alexa Fluor 647 anti-mouse TCR β chain clone H57-597 (BioLegend). Samples were then washed and analyzed on an Attune NxT Flow Cytometer, monitoring, as above, the percentage of cognate (CFSE^+^) and irrelevant (CFSE^-^) cells with surface-bound (Tetramer^+^MHC II^+^) versus internalized (Tetramer^+^MHC II^-^) aAPCs. SMART-A1 doped enrichment studies were performed analogously, except unlabeled SMART-A1 cells were used instead and detected with a PE anti-mouse CD45.1 clone A20 (Biolegend) antibody.

Doped enrichment and expansion studies were performed by diluting freshly isolated, unlabeled OT-II or SMART-A1 CD4^+^ T cells into recently isolated, unlabeled B6 CD4^+^ T cells. Cells were then incubated for 2 h with 30 ng conjugated I-A^b^/10^6^ CD4^+^ T cells of S1 aAPCs at 37 °C in T cell culture media and then magnetically enriched using a 96-well ring magnet^23^. The enriched fractions were plated at 2.5 × 10^5^ cells/mL in T cell culture media supplemented with Th1 skewing cytokines and 1 μg/mL soluble αCD28. Cells were refed on day 3 with half of the initial volume of T cell culture media and twice the concentration of cytokines. On day 7, the frequency and number of OT-II and SMART-A1 T cells were determined by harvesting and counting samples, staining them with tetramers or PE anti-mouse CD45.1 clone A20 (Biolegend) antibodies, respectively, and analyzing them on a BD FACSCalibur flow cytometer.

Endogenous murine CD4^+^ T cell enrichment and expansion studies were performed analogously to the doped enrichment and expansion studies, using freshly isolated B6 CD4^+^ T cells. On day 7, the frequency and number of antigen-specific CD4^+^ T cells was determined by harvesting and counting samples, staining them with cognate and non-cognate tetramers, and then analyzing them on a BD FACSCalibur flow cytometer.

Endogenous murine CD8^+^ T cell enrichment and expansion studies were performed as previously described in ref. 23, by isolating B6 CD8^+^ T cells, and then incubating them for 1 h with MHC I aAPCs (30 ng conjugated K^b^-Ig or D^b^-Ig per 10^6^ CD8^+^ T cells) at 4 °C in AutoMACS Running Buffer (1X PBS with 2 mM EDTA and 0.5% Bovine Serum Albumin). Cells were then magnetically enriched on a 96-well ring magnet and plated at 2.5 × 10^5^ cells/mL in T cell culture media supplemented with an optimized CD8^+^ cytokine mix^18^ and 1 μg/mL soluble αCD28. For endogenous coculture experiments, the enriched fractions were additionally supplemented with an equal number of Day 5 Th1-skewed CD4^+^ T cells (see above) and S1 aAPCs (80 ng/mL conjugated I-A^b^). Cells were refed on day 3 with half of the initial volume of T cell culture media and twice the concentration of the CD8^+^ cytokine mix. On day 7, cells were harvested and counted, and then analyzed for specificity, phenotype, and function of dimer positive CD8^+^ T cells. Representative gating strategies for binding, internalization, and enrichment experiments can be found in Supplementary Fig. 8a.

### Imaging studies

OT-I/OT-II imaging studies were performed by labeling freshly isolated OT-I CD8^+^ T cells at 37 °C for 20 min with 5 μM CellTracker green dye (Invitrogen) in T cell culture media without serum and then quenching at 37 °C for 5 additional minutes with 5 mL FBS. Analogously, freshly isolated or Day 5 Th1-skewed OT-II CD4^+^ T cells were labeled with 5 μM CellTrace Far Red dye (Invitrogen). Labeled OT-II CD4^+^ T cells were then preincubated with MHC I/II aAPCs at 80 ng conjugated I-A^b^/10^5^ CD4^+^ T cells for 2 h at 37 °C, prior to mixing them 1:1 with labeled OT-I CD8^+^ T cells. T cell mixtures were incubated on gelatin-coated (0.1%) plates and imaged using a Zeiss AxioObserver epifluorescent microscope with an incubation chamber at 37 °C and 5% CO_2_. Images at 24 h were analyzed using a custom protocol in CellProfiler. CD4^+^ and CD8^+^ T cells within five pixels of each other were considered bound.

OT-II internalization imaging studies were performed by incubating freshly isolated OT-II CD4^+^ T cells with nanoparticles conjugated with Alexa Fluor 488-labeled I-A^b^_OVA_ tetramer at a concentration of 80 ng I-A^b^/10^6^ cells for 2 h at 37 °C. Cells were then washed in PBS and stained with Alexa Fluor 594 anti-mouse CD4 clone GK1.5 (BioLegend) and Alexa Fluor 647 anti-mouse I-A/I-E clone M5/114.15.2 (BioLegend) antibodies for 15 min at 4 °C. Cells were then washed in PBS and fixed overnight in 1% paraformaldehyde. The following morning, cells were washed in PBS and stained with DAPI (Thermo Fisher) at 0.1 μg/mL for 10 min at 20 °C. Cells were then washed and imaged in a #1.5 chambered coverglass slide (Cellvis) using an LSM980 confocal microscope with Airyscan super-resolution. Airyscan processing was performed using Zen software, and the Pearson Correlation between Alexa Fluor 488 and Alexa Fluor 647 fluorescent signals was calculated in ImageJ.

### Transwell migration assays

Transwell migration assays were performed as previously described in ref. 54 using transwell plates (Costar) with 5.0 μm pore-size polycarbonate membranes. Day 7 stimulated OT-I CD8^+^ T cells were labeled at 37 °C for 20 min with 5 μM CFSE dye (Invitrogen) in T cell culture media without serum and then quenched at 37 °C for 5 additional minutes with 5 mL FBS. Analogously, freshly isolated or Day 5 Th1-skewed OT-II CD4^+^ T cells were labeled with 5 μM CellTrace Far Red dye (Invitrogen). The bottom compartments of the transwell plates received 600 μL of control medium (RPMI 1640 with 0.5% BSA) with or without 1 × 10^6^ labeled naïve or Th1 OT-II CD4^+^ T cells at a 1:1 ratio with αCD3/αCD28 Dynal microbeads, while the top compartments received 1 × 10^6^ OT-I CD8^+^ T cells in 100 μL control medium. Plates were incubated at 37 °C for 3 h and then the upper and lower compartments were harvested, manually counted with a hemocytometer, and stained with Live/Dead Fixable Violet (Invitrogen), PE anti-mouse CD4 clone H129.19 (BioLegend), and PE/Cyanine7 anti-mouse CD8 clone 53-6.7 (BD Biosciences). Cells were washed, resuspended in FACS Wash Buffer and analyzed on an Attune NxT Flow Cytometer. The transmigration index was calculated as the ratio of the number of CD8^+^ T cells transmigrated in a given sample to the number of CD8^+^ T cells transmigrated in the control medium.

### Protein arrays

Day 5 Th1 OT-II CD4^+^ T cells were either left unstimulated or were re-stimulated overnight with MHC II aAPCs (80 ng I-A^b^/10^5^ CD4^+^ T cells) and soluble αCD28 (1 μg/10^5^ CD4^+^ T cells). Cell supernatants were then collected and filtered through Spin-X Centrifuge Tube filters (Corning). Cytokines in the cell supernatants were then analyzed with the Proteome Profiler Mouse Cytokine Array Kit A (R&D Systems). The blots were visualized with chemiluminescence using an iBright 1500 imaging system and quantified using the Protein Array Analyzer plugin in ImageJ.

### Cloning of HA1.7 TCR

The native signal sequence and α and β variable domains of TCR HA1.7^55^ (IMGT ID 1FYT) were cloned into the AbVec mammalian expression vector^56^ containing the murine constant domains—to promote pairing of the exogenous α and β TCR chains—and human transmembrane domains (see Supplementary Table 2). The α and β chains were separated by a P2A peptide. The plasmid was purified using ZymoPURE II Plasmid Midiprep Kit (Zymo Research).

### HA1.7 expression and activation in Jurkat cells

10^7^ Jurkat cells per transfection were centrifuged at 250 × *g* for 5 min, resuspended in 5 mL of OptiMEM (Gibco), and incubated at 20 °C for 8 min. Cells were centrifuged as before, resuspended in 400 µl of OptiMEM and 20 µg HA1.7 plasmid, and transferred to a 4-mm electroporation cuvette (Bio-Rad). Cells were incubated for 8 min before pulsing exponentially with 250 V, 950 µF, and ∞ ohms resistance on a Bio-Rad GenePulser Xcell with PC and CE modules. After an 8 min recovery period, cells were rescued with 10 mL of prewarmed Jurkat culture media (RPMI 1640 + 10% FBS + 100 U/mL penicillin-streptomycin) and kept at 37 °C, 5% CO_2_.

In vitro stimulation of HA1.7 TCR-transfected Jurkat cells was performed 12–16 h after transfection. αCD3/αCD28 microbeads or titrations of nanoscale DR1 HA peptide exchanged and DR1 CLIP unexchanged aAPCs were incubated at 37 °C with 5 × 10^4^ transfected Jurkat T cells per stimulation in Jurkat culture media. At 24 h post-transfection, samples were washed and stained for 15 min at 4 °C in FACS Wash Buffer with APC anti-mouse TCR β chain clone H57-597 (BioLegend) and FITC anti-human CD69 clone FN50 (BioLegend) to detect the HA1.7 TCR and activation, respectively. Cells were then washed again and analyzed on a BD FACSCalibur Flow Cytometer.

### In vivo killing assay

One day prior to adoptive cell transfer (ACT), CD45.1 B6 mice received 500 cGy of irradiation to induce transient lymphopenia and promote T cell engraftment^57^. On the day of adoptive transfer, OT-II CD4^+^ T cells were either freshly isolated (naïve) or harvested after 7 days of stimulation with MHC II aAPCs (80 ng/mL conjugated I-A^b^ and 1 μg/mL soluble αCD28) in Th1 skewing media (Th1). Naïve and Th1 CD4^+^ T cells were labeled with 5 μM CellTrace Violet (CTV, Invitrogen) in 1 mL PBS for 20 min at 37 °C. The reaction was quenched with 5 mL FBS at 37 °C for 5 min, and then cells were washed twice in PBS. 10^6^ CTV labeled naïve or Th1 CD4^+^ T cells were then injected intravenously in volumes of 100 μL per recipient mouse. On the day of and the day after adoptive transfer, mice received intraperitoneal injections of 30,000 U IL-2 (Prometheus Labs) in a volume of 100 μL.

To analyze in vivo killing, 6 days post adoptive transfer, freshly isolated spleens from B6 mice were brought to a single cell suspension. Cells were then labeled either with 5 or 0.5 μM CFSE (Invitrogen) to generate CFSE^hi^ and CFSE^lo^ populations. CFSE^hi^ splenocytes were then loaded for 1 h at 37 °C with 1 μg of OVA_323-339_ peptide per 10^7^ cells in T cell culture media, washed twice in PBS, and mixed 1:1 with unloaded CFSE^lo^ splenocytes. 10^7^ cells of the mixture were then injected intravenously in 100 μL volumes per recipient mouse. The following day, spleens and lymph nodes of recipient mice were harvested, processed, and stained for Live/Dead Fixable Aqua (Invitrogen), PE anti-mouse CD45.2 clone 104 (BioLegend), APC anti-mouse CD4 clone GK1.5 (BioLegend), and PE/Cyanine7 anti-mouse I-A/I-E clone M5/114.15.2 (BioLegend) for 15 min at 4 °C. Cells were then washed, resuspended in FACS Wash Buffer, and analyzed on an Attune NxT Flow Cytometer. Specific lysis was calculated as 100% x (1-[(CFSE^lo,pre-injection^/CFSE^hi,pre-injection^)/(CFSE^lo,post-injection^/CFSE^hi,post-injection^)]). Representative gating strategies for in vivo killing assays can be found in Supplementary Fig. 8c.

To analyze in vivo phenotypic and functional markers, 21 days post adoptive transfer, spleens and lymph nodes were harvested from recipient mice, processed, resuspended at 10^7^ cells/mL in T cell culture media and incubated at 37 °C for 6 h with 1x cytokine activation cocktail (BioLegend) and GolgiPlug (BD Biosciences). No stimulation controls were received, only GolgiPlug. Following incubation, cells were washed and stained with Live/Dead Fixable Aqua (Invitrogen), PE anti-mouse CD45.2 clone 104 (BioLegend), and APC anti-mouse CD4 clone GK1.5 (BioLegend). Cells were then washed, fixed, and permeabilized overnight with the Foxp3 Transcription Factor Staining Buffer Set (eBioscience), and then stained with APC anti-mouse IFN-γ clone XMG1.2 (BioLegend), PE/Cyanine7 anti-mouse TNF-α clone MP6-XT22 (BioLegend), and PerCp-Cyanine5.5 anti-mouse/human T-bet clone eBio4B10 (eBioscience) or its corresponding isotype. Finally, cells were washed and resuspended in FACS wash buffer (1X PBS, 2% FBS, 0.5% sodium azide) and then analyzed on an Attune NxT Flow Cytometer.

### Adoptive transfer melanoma model

The in vivo therapeutic efficacy of OT-I CD8^+^ T cells cocultured with Th1 OT-II CD4^+^ T cells was compared to traditionally stimulated OT-I CD8^+^ T cells using a B16-OVA murine melanoma model. On day 0, B6 mice received a subcutaneous injection of 2 × 10^5^ tumor cells on the left flank. On that same day, OT-II CD4^+^ T cells were activated in Th1 skewing media with MHC II aAPCs (80 ng/mL conjugated I-A^b^ and 1 μg/mL soluble αCD28). On day 5, OT-I CD8^+^ T cells were stimulated with MHC I aAPCs (30 ng/mL conjugated K^b^ and 1 μg/mL soluble αCD28) in T cell culture media supplemented with TF. Cocultured OT-I CD8^+^ T cells were additionally mixed at a 1:1 ratio with the day 5 Th1 OT-II CD4^+^ T cells and MHC II aAPCs (80 ng/mL conjugated I-A^b^). On day 10, 2 × 10^6^ OT-I CD8^+^ T cells that were freshly isolated, stimulated alone, or stimulated in coculture with Th1 OT-II CD4^+^ T cells, were injected intravenously in 100 μL volumes into B16-OVA tumor-bearing mice. On the day of and the day after adoptive transfer, mice received intraperitoneal injections of 30,000 U IL-2 (Prometheus Labs) in 100 μL volumes. Tumor size was measured with digital calipers every 2–3 days until tumors became necrotic or reached 200 mm^2^, after which mice were sacrificed with CO_2_ asphyxiation and cervical dislocation.

### Statistical analysis

Error bars in graphs represent the standard error of the mean (s.e.m.) unless otherwise stated. All *n* values are given in the Figure legends. Statistical analyses were performed in GraphPad Prism software version 8.4.3. Two-tailed Student’s *t*-tests were used for comparisons between the two groups. One and two-way ANOVAs with Tukey’s multiple-comparisons test were used for comparisons between multiple groups. One-way ANOVAs with Dunnett’s post hoc test was used for comparison of multiple groups to a control group. Repeated measure two-way ANOVAs with Tukey’s multiple-comparisons test were used for comparing tumor growth curves, and log-rank tests were used for comparing survival curves.

### Reporting summary

Further information on research design is available in the Nature Research Reporting Summary linked to this article. The data supporting the findings in this study are available in either the main manuscript or supplementary figures. Life Science and Flow Cytometry Reporting Summaries are available in the Nature Research Reporting Summary linked to this article.

## Supplementary information

Supplementary Information

Reporting Summary

## Data Availability

Raw data used for figures and plots are provided along with this paper as a Source Data file. Any additional datasets generated and/or analyzed for this manuscript are available from the corresponding author upon reasonable request. Source data are provided with this paper.

## Source data

Source Data
