# Supplementary material for: Nanoparticle-based modulation of CD4+ T cell effector and helper functions enhances adoptive immunotherapy
Source: Nat Commun. 2022 Oct 14;13:6086. doi: 10.1038/s41467-022-33597-y (PMC9568616; doi:10.1038/s41467-022-33597-y)
Supplement: Supplementary file 1 — Supplementary Information [file 41467_2022_33597_MOESM1_ESM.pdf]

SUPPLEMENTARY INFORMATION

Nanoparticle-based modulation of CD4$^+$ T cell effector and helper functions enhances adoptive immunotherapy

*Ariel Isser[1,2], Aliyah B. Silver[2,3,4], Hawley C. Pruitt[5,6], Michal Mass[1,4], Emma H. Elias[7], Gohta Aihara[1], Si-Sim Kang[8], Niklas Bachmann[8], Ying-Yu Chen[8], Elissa K. Leonard[1,2,4], Joan G. Bieler[2,8], Worarat Chaisawangwong[2,8], Joseph Choy[2,4,6,9], Sydney R. Shannon[1,2,4], Sharon Gerecht[1,5,6,9,10], Jeffrey S. Weber[11], Jamie B. Spangler[1,4,5,9,10,12,13], and Jonathan P. Schneck[1,2,8,10,14]**

[1]Department of Biomedical Engineering, Johns Hopkins University School of Medicine, Baltimore, MD, 21287, USA
[2]Johns Hopkins Translational ImmunoEngineering Center, Johns Hopkins University School of Medicine, Baltimore, MD, 21287, USA
[3]Department of Molecular Microbiology and Immunology, Johns Hopkins University Bloomberg School of Public Health, Baltimore, MD, 21287, USA
[4]Translational Tissue Engineering Center, Johns Hopkins University School of Medicine, Baltimore, MD, 21287, USA
[5]Department of Chemical and Biomolecular Engineering, Johns Hopkins University Whiting School of Engineering, Baltimore, MD, 21287, USA
[6]Institute for NanoBioTechnology, Johns Hopkins University Whiting School of Engineering, Baltimore, MD, 21287, USA
[7]Department of Biology, Johns Hopkins University Krieger School of Arts and Sciences, Baltimore, MD, 21287, USA
[8]Department of Pathology, Johns Hopkins University School of Medicine, Baltimore, MD, 21287, USA
[9]Department of Materials Science and Engineering, Johns Hopkins University Whiting School of Engineering, Baltimore, MD, 21287, USA
[10]Department of Oncology, Johns Hopkins University School of Medicine, Baltimore, MD, 21287, USA
[11]Laura and Isaac Perlmutter Cancer Center, NYU Langone Health, New York, New York, 10016, USA
[12]Department of Ophthalmology, Wilmer Eye Institute, Johns Hopkins University School of Medicine, Baltimore, MD, 21287, USA
[13]Bloomberg~Kimmel Institute for Cancer Immunotherapy, Sidney Kimmel Comprehensive Cancer Center, Johns Hopkins University School of Medicine, Baltimore, MD, 21287, USA
[14]Institute for Cell Engineering, Johns Hopkins University School of Medicine, Baltimore, MD, 21287, USA

*Correspondence: jschnec1@jhmi.edu

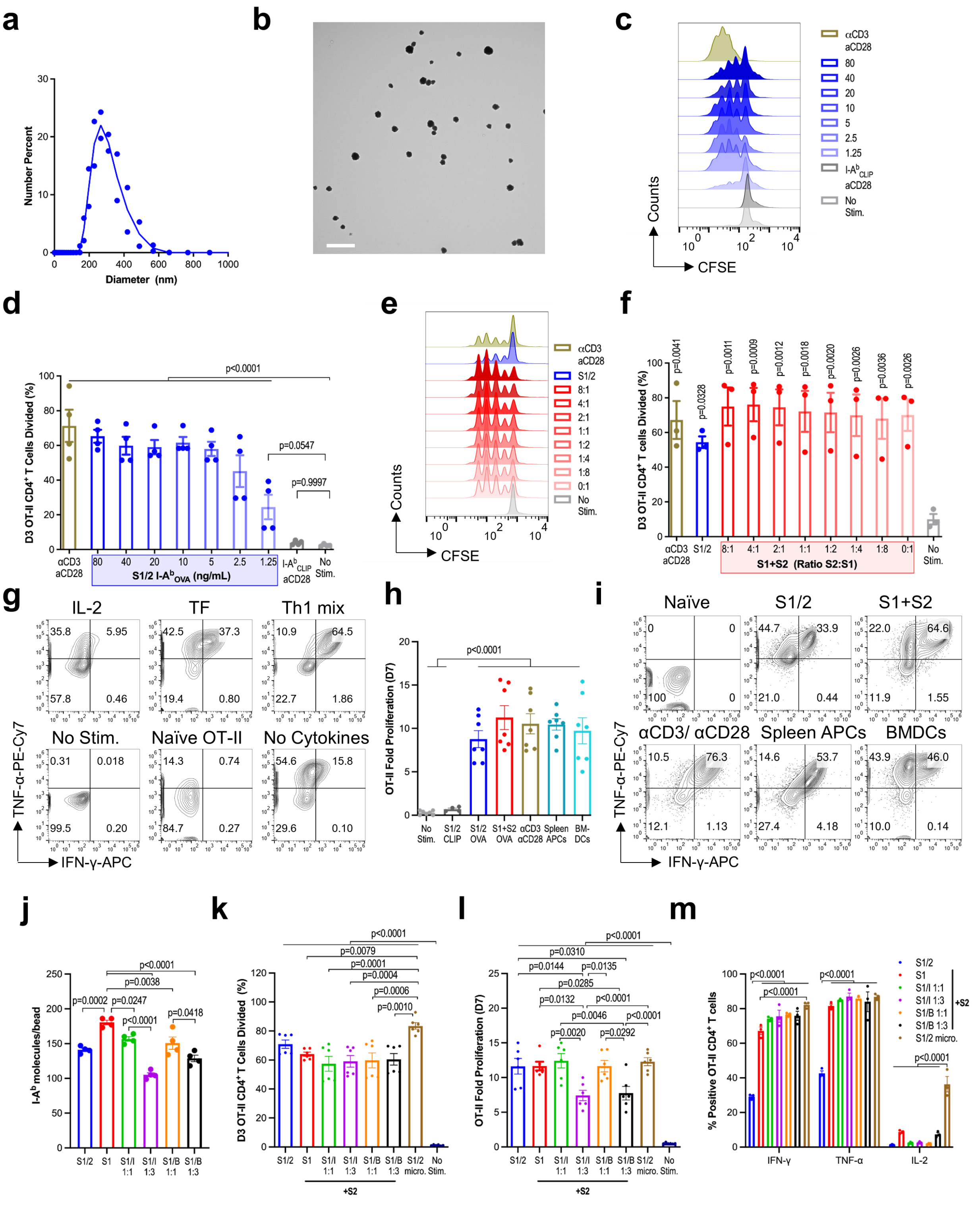

Supplementary Figure 1: Characterization and function of MHC II aAPCs. (**a**) Size distribution of MHC II aAPCs as measured by dynamic light scattering (DLS). (**b**) Transmission electron imaging of MHC II aAPCs. Scale bar: 500 nm. (**c**) CFSE dilutions and

(**d**) percentage of OT-II $CD4^+$ T cells divided after 3 days of stimulation with a titration of $I\text{-}A^b{}_{OVA}$ S1/2 aAPCs compared to polyclonal αCD3/αCD28 or $I\text{-}A^b{}_{CLIP}$ aAPCs. (**e**) CFSE dilutions and (**f**) percentage of OT-II cells divided after 3 days of stimulation with $I\text{-}A^b{}_{OVA}$ S1 aAPCs and a titration of S2, compared to S1/2 or αCD3/αCD28 aAPCs. (**g**) Representative day 7 cytokine staining of OT-II cells stimulated with S1/2 aAPCs in media containing: no cytokines, IL-2, T cell growth factor (TF) cytokine cocktail, or a Th1 mix (IL-2, IL-12p70, IFN-γ). (**h-i**) Fold proliferation and representative day 7 cytokine staining of OT-II cells stimulated with saturating doses of S1/2, S1+S2, or αCD3/αCD28 aAPCs versus peptide pulsed OT-II splenocytes or bone-marrow derived dendritic cells (BMDCs). (**j**) Fluorescent quantification of $I\text{-}A^b{}_{OVA}$ on 300 nm nanoparticles conjugated with S1, S1 and αCD28 (S1/2) at a 1:1 ratio, S1 and isotype antibodies (S1/I), or S1 and BSA (S1/B) at 1:1 and 1:3 ratios. (**k**) Day 3 CFSE, (**l**) day 7 fold proliferation, and (**m**) day 7 cytokine secretion of OT-II $CD4^+$ T cells stimulated with S1/2, S1, S1/I, and S1/B nanoparticles with soluble S2, or S1/2 4.5 μm microparticles. Data in (**a-b**) are representative of two independent samples. Data in (**d,f,h,j-m**) represent mean ± standard error of the mean (s.e.m.) from three or more independent experiments. (**d**) n = 4 mice, (**f**) n = 3 mice, (**h**) n = 4 (CLIP) or 7 (No Stim., Spleen APCs, BMDCs, αCD3/αCD28, S1/2, S1+S2) mice, (**i**) n = 4, (**j-k**) n = 6 mice, (**l**) n = 3 mice, analyzed using a (**d,f**) one-way ANOVA compared to no stim. condition with Dunnet's multiple-comparisons test, an (**h,j-l**) ordinary one-way, or a (**m**) two-way ANOVA with Tukey's multiple-comparisons test.

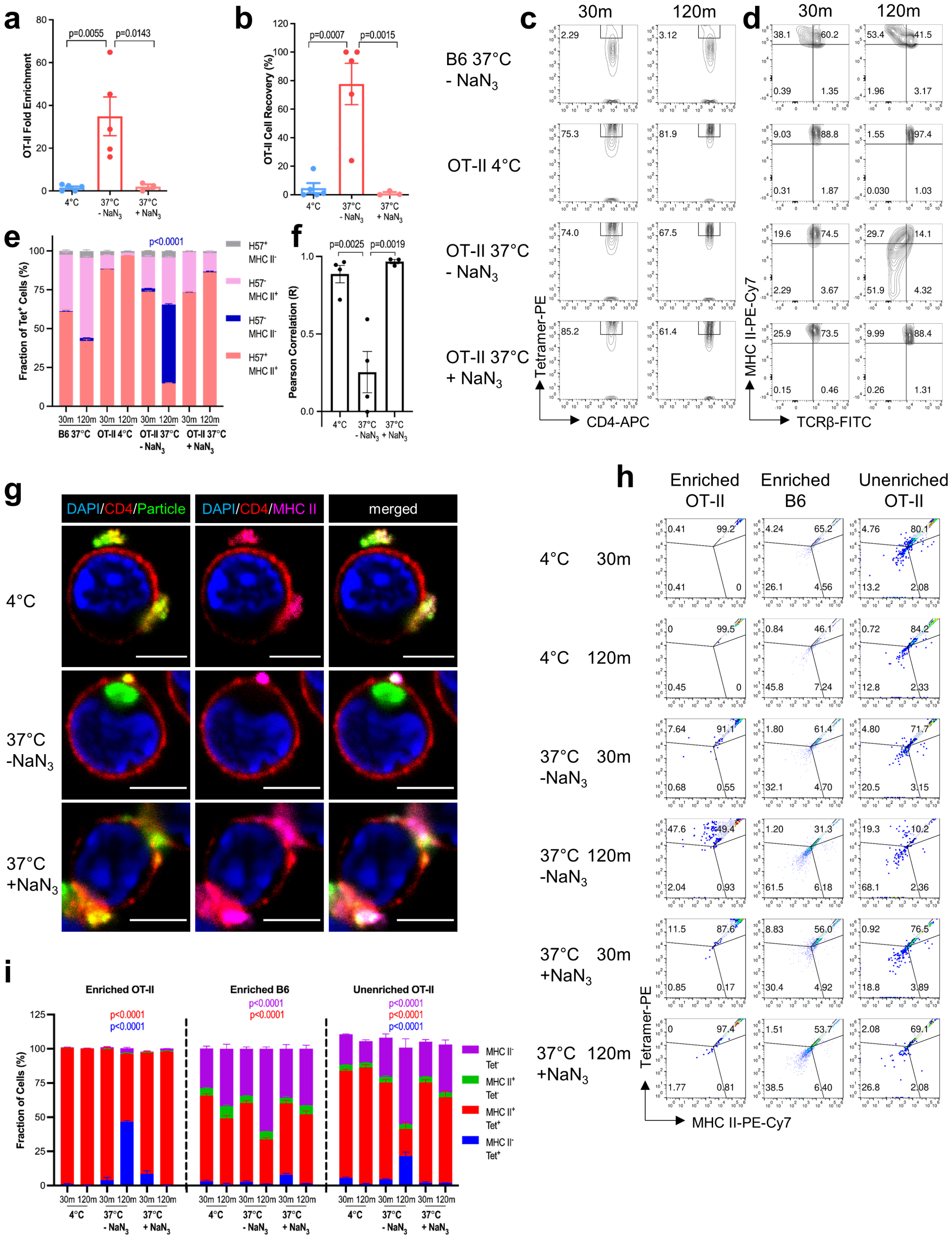

Supplementary Figure 2: Antigen-specific MHC II aAPC internalization enhances $CD4^+$ T cell magnetic enrichment. (**a**) Fold enrichment and (**b**) percent cell recovery of

OT-II cells diluted into a B6 background at a ratio of 1:1000 after magnetic enrichment with S1 aAPCs after 2 hours of incubation at various temperatures with and without sodium azide (NaN_3) uptake inhibitor. (**c-e**) Binding and internalization of PE-labelled S1 aAPCs by OT-II (cog.) and B6 (non-cog) $CD4^+$ T cells after incubation for 30 minutes and 2 hours at various temperatures with and without NaN_3. $CD4^+$ T cells with particles on their surface are $Tet^+MHC\ II^+$, whereas cells with internalized particles are $Tet^+MHC\ II^-$. (**c**) Representative flow plots of $CD4^+$ T cells that have either bound or internalized particles, (**d**) Representative flow plots and (**e**) overall MHC II and TCRβ staining of the Tet^+ $CD4^+$ T cells from (**c**). (**f**) Pearson's correlation of MHC II detection and particle fluorescence from (**g**) confocal imaging of OT-II $CD4^+$ T cells incubated with AF488-labelled S1 aAPCs after incubation for 2 hours at various temperatures with and without NaN_3. Scale bar: 4 μm. (**h-i**) Particle internalization tracking after magnetic enrichment of OT-II cells diluted into a B6 background at a ratio of 1:1000 with PE-labelled S1 aAPCs after incubation for 30 minutes and 2 hours at various temperatures with and without NaN_3. (**h**) Representative flow plots and (**i**) overall MHC II and PE staining of enriched OT-II, enriched B6, or unenriched OT-II $CD4^+$ T cell populations from the enrichment experiments. Data in (**a-b, e-f, i**) represent mean ± standard error of the mean (s.e.m.) from three or more independent experiments. (**a**) n = 3 (37 ° C+NaN_3) or 5 (4 ° C, 37 ° C-NaN_3) mice, (**e**) n = 3 mice, (**f**) n = 3 (37 ° C+NaN_3) or 4 (4 ° C, 37 ° C-NaN_3), (**i**) n = 3 mice, analyzed using a one-way (**a-b,f**) or two-way ANOVA (**e,i**) with Tukey's multiple-comparisons test.

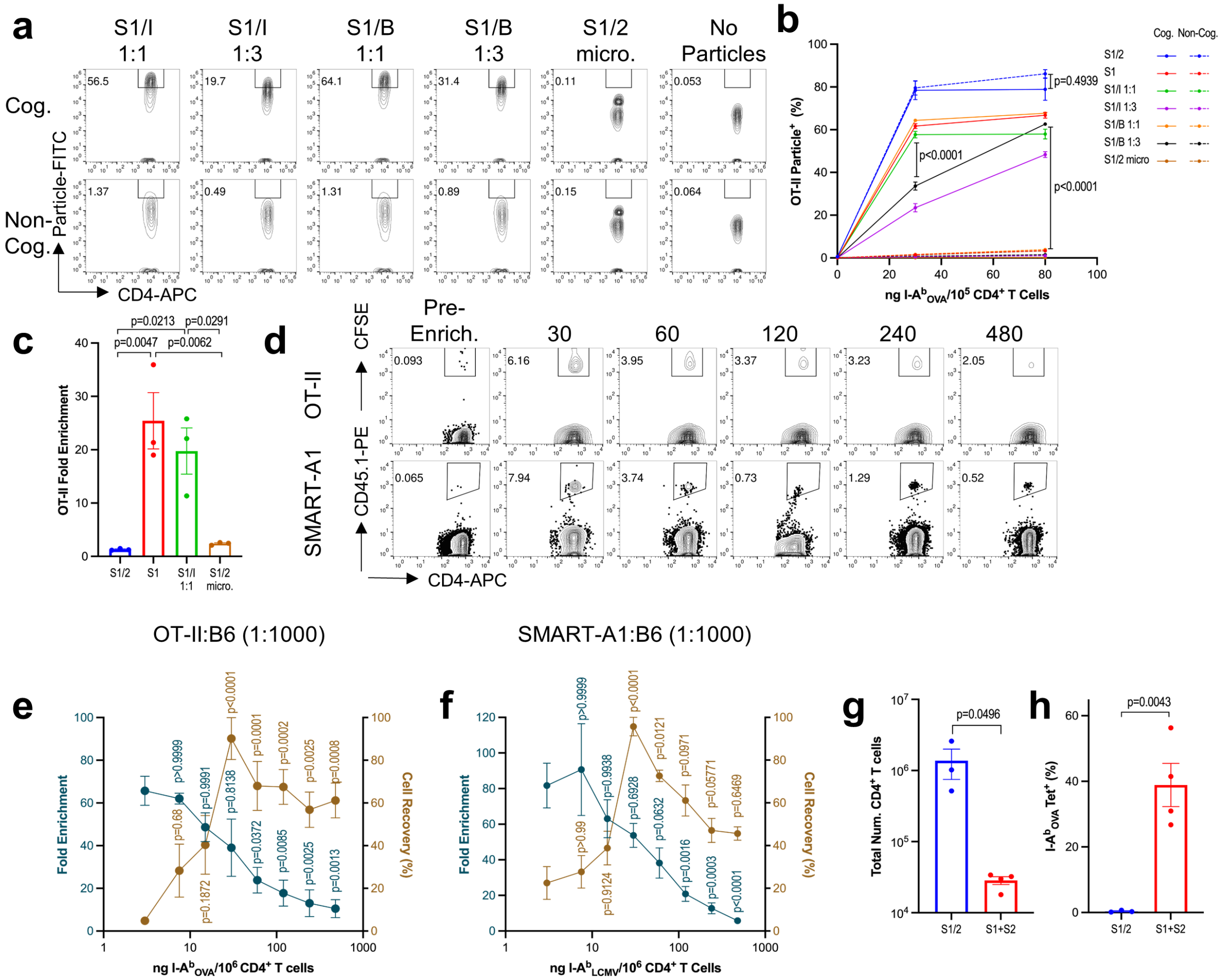

Supplementary Figure 3: Impact of MHC II aAPC size, ligand density, and dosing on antigen-specific CD4$^+$ T cell binding and enrichment. (**a-b**) Particle binding to OT-II (cog.) and B6 (non-cog.) CD4$^+$ T cells after incubation at 30 minutes and 37°C with 300 nm nanoparticles conjugated with S1, S1 and αCD28 (S1/2) at a 1:1 ratio, S1 and isotype antibodies (S1/I) or BSA (S1/B) at 1:1 or 1:3 ratios, or with S1/2 4.5 μm microparticles. (**a**) Representative flow plots at 30 ng I-A^b/10^5 CD4$^+$ T cells, and (**b**) Percent cells bound across a range of doses. (**c**) OT-II CD4$^+$ T cells were diluted 1:1000 into a B6 background and incubated for 2 hours at 37°C with 30 ng I-A^b/10^6 CD4$^+$ T cells of S1/2, S1, or S1/I 1:1 nano-aAPCs versus S1/2 micro-aAPCs. Fold enrichment of magnetically enriched samples relative to baseline. (**d**) Representative flow plots of OT-II (top) and

SMART-A1 $CD4^+$ T cells (bottom) pre and post-enrichment, (**e-f**) fold enrichment and percent cell recovery of (**e**) OT-II and (**f**) SMART-A1 cells post-enrichment with a titration of cognate S1 nano-aAPCs. (**g**) Total number of $CD4^+$ T cells and (**h**) percentage of $I\text{-}A^b_{OVA}$ tetramer positive $CD4^+$ T cells 7 days after S1/2 or S1+S2 enrichment and expansion. Data in (**b,c,e-h**) represent mean ± standard error of the mean (s.e.m.) from three or more independent experiments. (**b-c**) n = 3 mice, (**e**) n = 3 (3-30 ng) or 5 (60-480 ng) mice, (**f**) n = 3 mice, and (**g-h**) n=3 (S1/2) or 4 (S1+S2) mice analyzed using a (**b**) two-way or (**c,e-f**) one-way ANOVA with Tukey's multiple-comparisons test, or (**g-h**) an unpaired Student's *t* test.

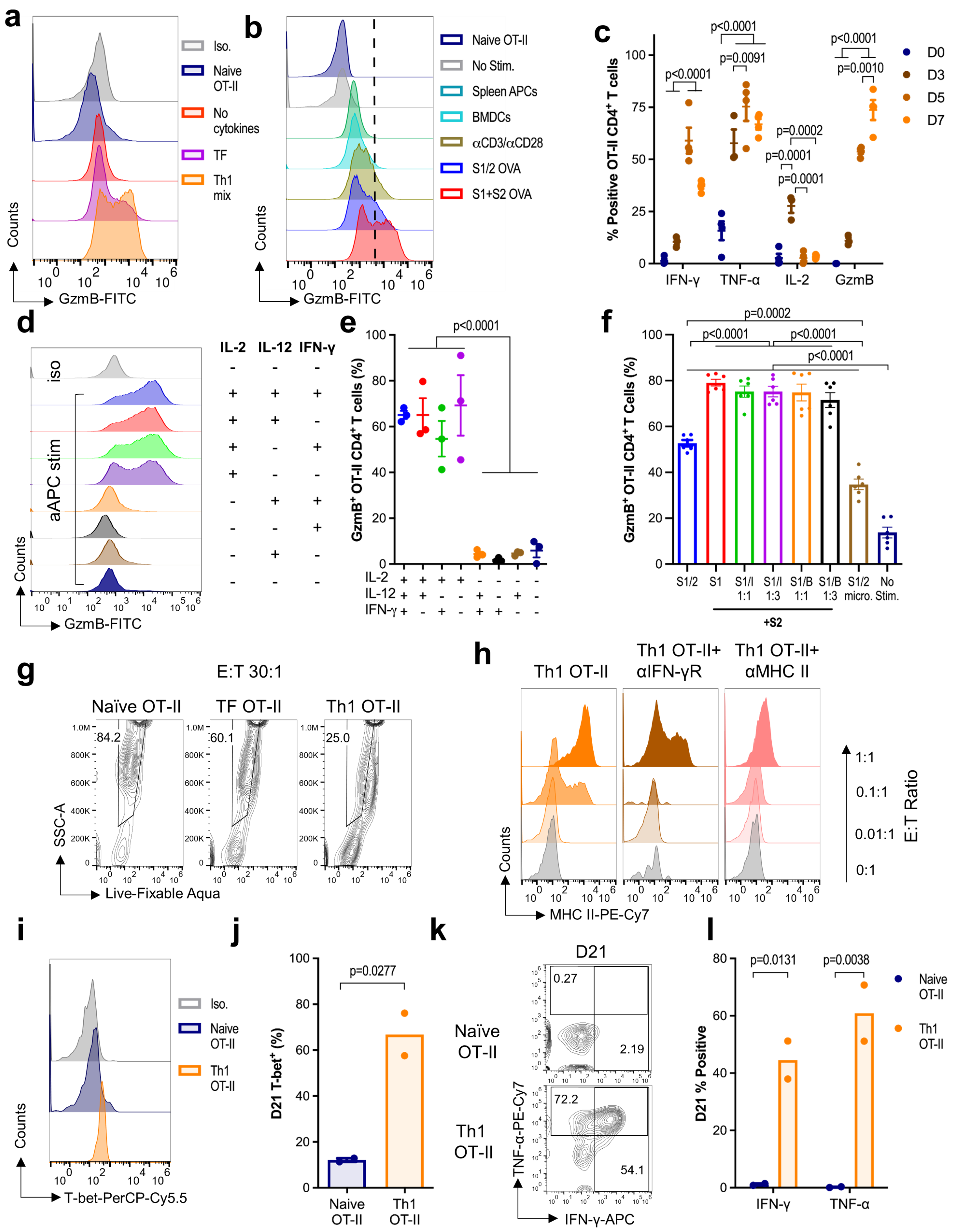

Supplementary Figure 4: MHC II aAPCs promote CD4^{+} T cell cytotoxicity. (**a**) Day 7 GzmB levels in OT-II cells stimulated with S1+S2 aAPCs in media containing: no cyto-

kines, TF, or a Th1 mix (IL-2, IL-12p70, IFN-γ). (**b**) Day 7 GzmB levels of OT-II cells stimulated in Th1 media with S1/2, S1+S2, or αCD3/αCD28 aAPCs versus peptide pulsed OT-II splenocytes or bone-marow derived dendritic cells (BMDCs). (**c**) OT-II cytokine production on days 0, 3, 5, 7 of stimulation with S1 *a*APCs in Th1 media. (**d**) GzmB staining and (**e**) percent positive of OT-II cells after 7 days of S1+S2 stimulation in the various components of the Th1 mix. (**f**) Percentage of $GzmB^+$ OT-II cells after 7 days of stimulation with 300 nm nanoparticles conjugated with S1, S1 and αCD28 (S1/2) at a 1:1 ratio, S1 and isotype antibodies (S1/I) or BSA (S1/B) at 1:1 or 1:3 ratios, or S1/2 4.5 μm microparticles. (**g**) B16-OVA tumor cell viability after overnight incubation at an effector-to-target (E:T) ratio of 30:1 with naïve or *a*APC stimulated OT-II cells cultured in TF or Th1 media. (**h**) Live B16-OVA MHC II expression after overnight incubation with aAPC stimulated Th1 OT-II cells and MHC II or IFN-γR antibody blocking. (**i**) T-bet staining and (**j**) percentage, (**k***)* IFN-γ and TNF-α staining and (**l**) percentage of naïve versus Th1 OT-II $CD4^+$ T cells 21 days post adoptive cell transfer (ACT). Data in (**c,e,f,j,l**) represent mean ± standard error of the mean (s.e.m.). (**c**) n = 3 (D3) or 4 (D0, D5, D7) mice, (**e**) n = 3 mice, (**f**) n =6 mice, analyzed using a (**c**) two-way or (**e-f**) one-way ANOVA with Tukey's multiple-comparisons test, (**j,l**) n = 2 mice/group analyzed using an (**j**) unpaired Student's *t* test, two-tailed or (**l**) two-way ANOVA with Tukey's multiple-comparisons test.

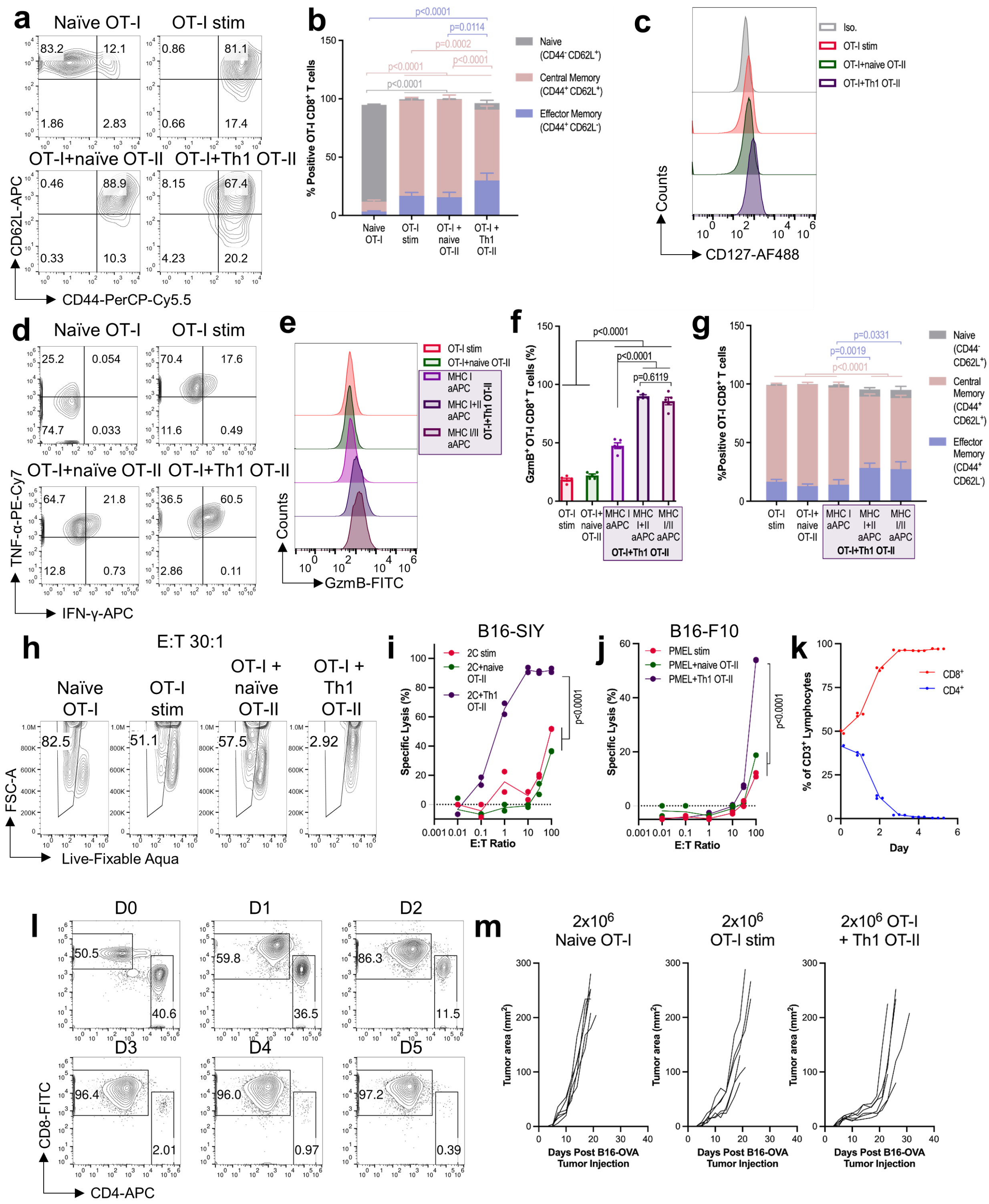

Supplementary Figure 5: MHC II aAPCs modulate $CD4^+$ T cell helper function. (**a-d**) OT-I cells in TF supplemented media were activated with MHC I K^b_{OVA} aAPCs either alone or in co-culture with naïve or aAPC activated Th1 OT-II cells and MHC II $I\text{-}A^b_{OVA}$

aAPCs. Day 7 (**a-b**) memory phenotype, (**c**) CD127 expression, and (**d**) cytokine staining from the various stimulations. (**e-f**) OT-I cells were cultured as above but with different stimuli: K^{b}_{OVA} only (MHC I), separate (MHC I+II), and co-presenting (MHC I/II) aAPCs. Day 7 (**e-f**) intracellular GzmB levels and (**g**) memory phenotype of OT-I cells stimulated under these various conditions. (**h**) B16-OVA viability after overnight incubation at an E:T ratio of 30:1 with OT-I cells stimulated alone or co-cultured with naïve or Th1 OT-II cells. (**i**) B16-SIY and (**j**) B16-F10 specific lysis after overnight incubation with 2C or PMEL $CD8^{+}$ T cells, respectively, stimulated alone or co-cultured with naïve or Th1 OT-II cells. (**k-l**) Percentage of $CD3^{+}$ lymphocytes that are $CD4^{+}$ or $CD8^{+}$ T cells over five days of OT-I and Th1 OT-II co-culture. (**m**) Spider plots depicting tumor growth of B16-OVA in B6 mice subjected to adoptive transfer of OT-I cells that were either freshly isolated, activated alone, or co-activated with Th1 OT-II $CD4^{+}$ T cells. Data in (**b,f,g,i-k**) represent mean ± standard error of the mean (s.e.m.) from two or more independent experiments. (**b**) n = 4 mice, (**f**) n = 5 mice, (**g**) n = 3 (MHC I/II) or 6 (OT-I stim, OT-I+naïve OT-II, MHC I, MHC I+II) mice, (**i-j**) n = 2 mice, and (**k**) n = 3 mice, analyzed using a (**f**) one-way or (**b,g**) two-way ANOVA with Tukey's multiple-comparisons test.

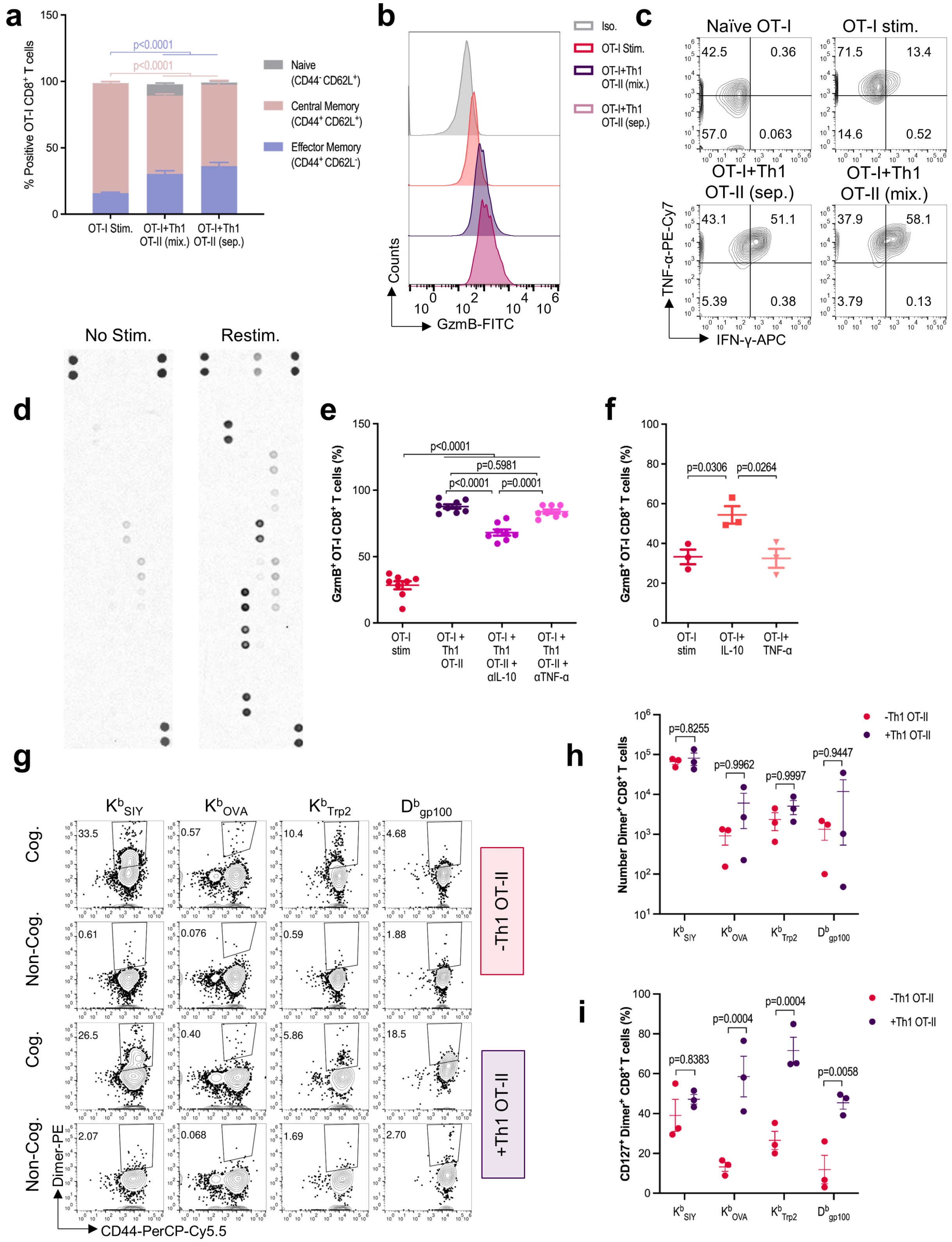

Supplementary Figure 6: aAPC mediated T cell help is driven by soluble factors and extends to endogenous CD8+ T cells. (**a**) Memory phenotype, (**b**) intracellular GzmB

levels, and (**c**) cytokine staining of OT-I cells activated alone, separated (sep.) from, or mixed (mix.) with Th1 OT-II cells in a transwell plate. (**d**) Representative cytokine arrays of supernatants harvested from unstimulated or re-stimulated Th1 OT-II cells. (**e**) Flow cytometry detection of GzmB expression in OT-I cells co-cultured with Th1 OT-II cells in the presence of blocking antibodies to IL-10 and TNF-α. (**f**) Flow cytometry detection of GzmB in OT-I cells stimulated in media supplemented with IL-10 or TNF-α. (**g-i**) K^{b}_{SIY}, K^{b}_{OVA}, K^{b}_{Trp2}, and D^{b}_{gp100} specific $CD8^{+}$ T cells were enriched from B6 mice and then expanded either alone or in co-culture with Th1 OT-II cells. (**g**) Dimer staining and (**h**) numbers of $CD8^{+}$ T cells of corresponding antigenic specificities at day 7. (**i**) Percent of antigen-specific $CD8^{+}$ T cells that were CD127 positive. Data in (**a,e-f,h-i**) represent mean ± standard error of the mean (s.e.m.) and three or more independent experiments. (**a**) n = 3 mice, (**e**) n = 8 mice, (**f**) n = 3 mice, and (**h-i**) n = 3 mice analyzed using a (**e-f,h-i**) one-way or (**a**) two-way ANOVA with Tukey's multiple-comparisons test.

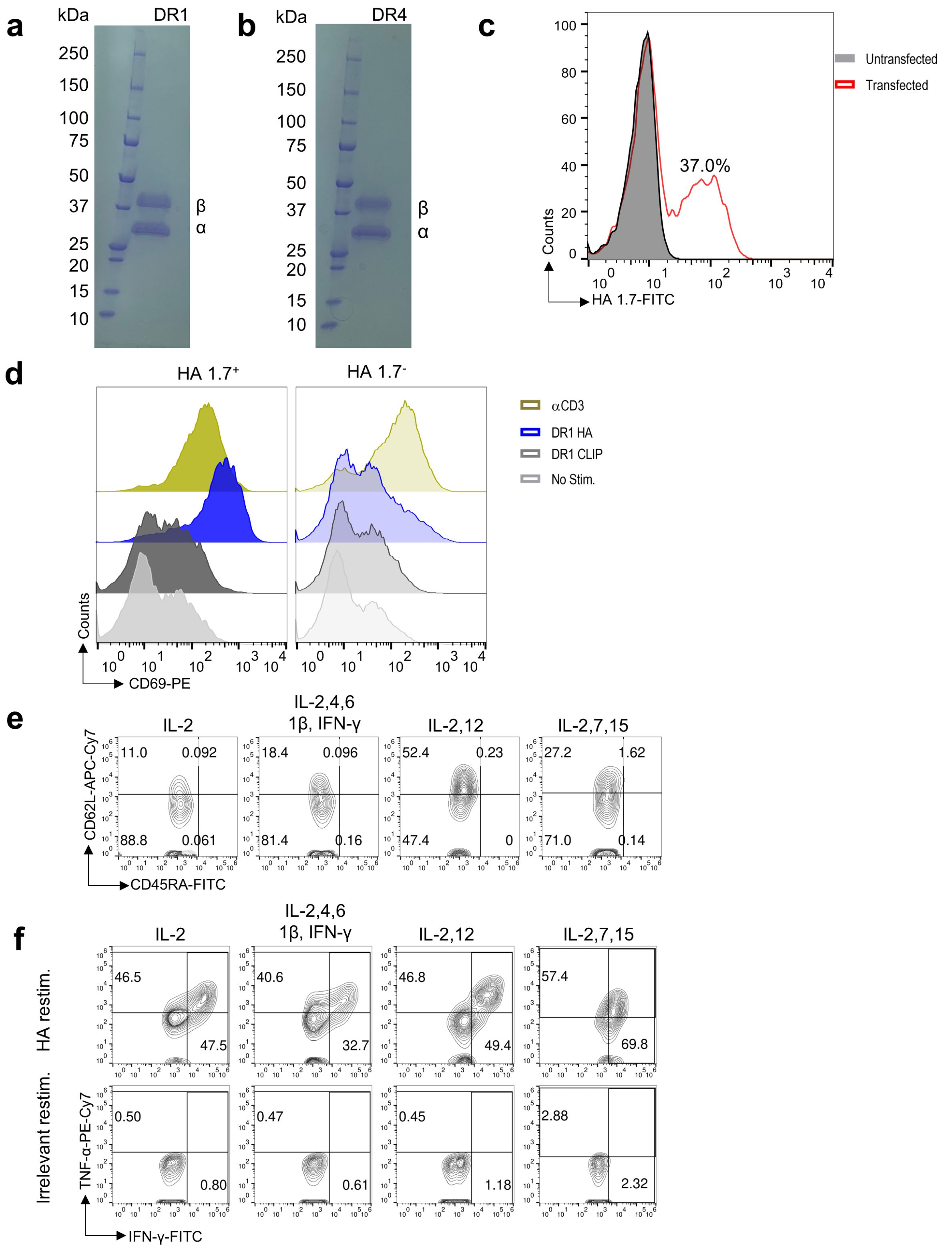

Supplementary Figure 7: HLA II aAPCs stimulate functional antigen-specific human CD4+ T cells. (**a-b**) SDS-PAGE analysis of human embryonic kidney (HEK) 293-F cell-

secreted (**a**) DR1 and (**b**) DR4 monomers. (**c**) Detection of HA 1.7 TCR on Jurkat cells after overnight transfection and (**d**) comparison of CD69 induction on HA1.7 TCR positive and negative Jurkat cells following stimulation with either αCD3/αCD28 microparticles or a titration of DR1/αCD28 aAPCs loaded with cognate hemagglutinin (DR1 HA) or non-cognate CLIP (DR1 CLIP) peptides. (**e**) Memory phenotype and (**f**) intracellular cytokine production after cognate (HA) and irrelevant (NY-ESO-1) peptide stimulation of DR4 HA tetramer positive $CD4^+$ T cells expanded from healthy donor peripheral blood mononuclear cells (PBMC) using DR4 HA aAPCs and four cytokine mixes: (i) IL-2 only; (ii) IL-2, IL-4, IL-6, IL-1β, and IFN-γ; (iii) IL-2 and 12; and (iv) IL-2, IL-7, and IL-15.

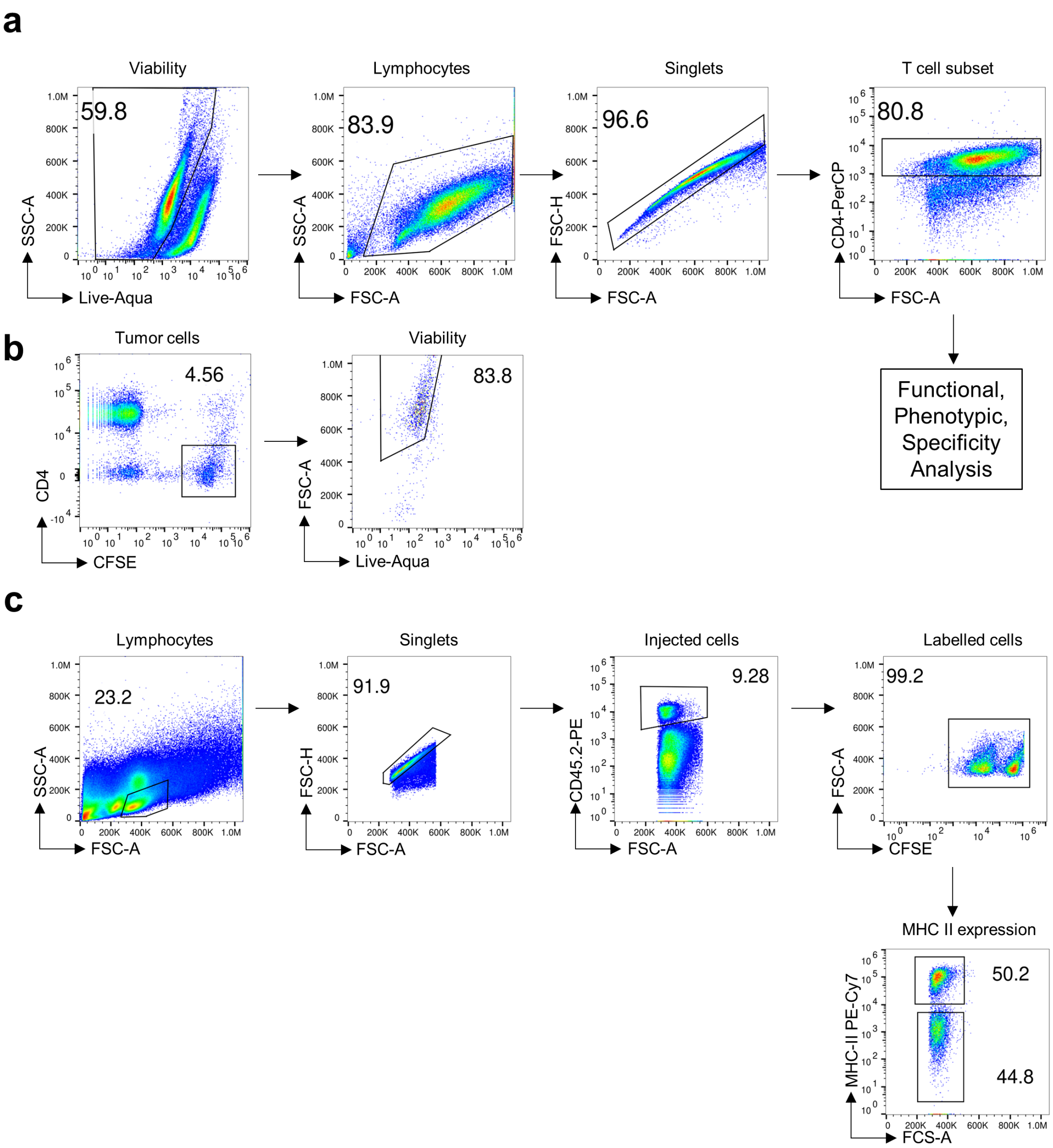

Supplementary Figure 8: Representative flow cytometry gating strategies. (**a**) Gating strategy for T cell functional, phenotypic, or specificity analysis, based on sequential gating for viability markers, lymphocytes, singlets, and then T cell subsets. (**b**) Gating strategy for *in vitro* killing assays, based on sequential gating for CFSE labelled tumor cells and viability markers. (**c**) Gating strategy for *in vivo* killing assays, based on sequential gating on lymphocytes, singlets, CD45.2 and CFSE positive cells, and then either MHC II high or low subsets.

Supplementary Table 1. Antibody List

Antibody	Amount	Clone	Source	Cat. #	Validation
anti-mouse CD3e	100 ug/mg beads	145-2C11	BioXcell	BE0001-1	T cell activation
anti-mouse CD28	1 ug/mL	37.51	BioXcell	BE0015-1	T cell activation
anti-mouse OX40	1 ug/mL	OX-86	BioXcell	BE0031	T cell activation
anti-mouse IFNγR	10 ug/mL	GR-20	BioXcell	BE0029	IFNγR neutralization
anti-mouse I-A/I-E	10 ug/mL	M5/114	BioXcell	BE0108	MHC II blockade
anti-mouse TNFα	1 ug/mL	XT3.11	BioXcell	BE0058	TNFα neutralization
anti-mouse IL-10	1 ug/mL	JES5-2A5	BioXcell	BE0049	IL-10 neutralization
anti-human CD3	100 ug/mg beads	OKT-3	BioXcell	BE0001-2	T cell activation
anti-human CD28	1 ug/mL	9.3	BioXcell	BE0248	T cell activation
PE anti-mouse CD3	1:100	17A2	BioLegend	100206	Flow Cytometry
APC anti-mouse CD4	1:100	GK1.5	BioLegend	100412	Flow Cytometry
PerCP anti-mouse CD4	1:100	RM4-5	BioLegend	100538	Flow Cytometry
PE anti-mouse CD4	1:100	H129.19	BioLegend	130310	Flow Cytometry
APC-Cyanine7 anti-mouse CD4	1:100	GK1.5	BioLegend	100414	Flow Cytometry
Alexa Fluor 594 anti-mouse CD4	1:100	GK1.5	BioLegend	100446	Flow Cytometry
APC anti-mouse CD8a	1:100	53-6.7	BioLegend	100712	Flow Cytometry
PerCP anti-mouse CD8	1:100	53-6.7	Biolegend	100732	Flow Cytometry

APC/Cyanine 7 anti-mouse CD8a	1:100	53-6.7	BioLegend	100714	Flow Cytometry
PE/Cyanine7 anti-mouse CD8	1:100	53-6.7	BD Biosciences	561097	Flow Cytometry
PerCP-Cy5.5 anti-mouse CD44	1:100	IM7	BioLegend	103032	Flow Cytometry
PE anti-mouse CD45.1	1:100	A20	Biolegend	110708	Flow Cytometry
PE anti-mouse CD45.2	1:100	104	BioLegend	109808	Flow Cytometry
APC anti-mouse CD62L	1:100	MEL-14	BioLegend	104412	Flow Cytometry
Alexa Fluor 488 anti-mouse CD127	1:100	A7R34	BioLegend	135018	Flow Cytometry
PE/Cyanine7 anti-mouse CD197 (CCR7)	1:100	4B12	BioLegend	120124	Flow Cytometry
Brilliant Violet 605 anti-mouse/human KLRG1	1:100	2F1/KLRG 1	BioLegend	138419	Flow Cytometry
FITC anti-mouse I-A/I-E	1:100	M5/114.15 .2	BioLegend	107606	Flow Cytometry
Alexa Fluor 647 anti-mouse I-A/I-E	1:100	M5/114.15 .2	BioLegend	107618	Flow Cytometry
PE/Cyanine7 anti-mouse I-A/I-E	1:100	M5/114.15 .2	BioLegend	107630	Flow Cytometry
FITC anti-mouse TCR β chain	1:100	H57-597	BioLegend	109206	Flow Cytometry
APC anti-mouse TCR β chain	1:100	H57-597	BioLegend	109212	Flow Cytometry
Alexa Fluor 647 anti-mouse TCR β chain	1:100	H57-597	BioLegend	109218	Flow Cytometry
FITC anti-mouse Foxp3	1:100	FJK-16s	eBioscience	14-5773-82	Flow Cytometry
PerCP-Cyanine5.5 anti-mouse/human T-bet	1:100	eBio4B10	eBioscience	45-5825-82	Flow Cytometry
APC anti-mouse/human RORγT	1:100	AFKJS-9	eBioscience	17-6988-82	Flow Cytometry

PE/Cyanine7 anti-mouse/human Gata3	1:100	TWAJ	eBioscience	25-9966-42	Flow Cytometry
APC anti-mouse IFN-γ	1:100	XMG1.2	BioLegend	505810	Flow Cytometry
PE/Cyanine7 anti-mouse TNF-α	1:100	MP6-XT22	BioLegend	506324	Flow Cytometry
PE anti-mouse IL-2	1:100	JES6-5H4	BioLegend	503808	Flow Cytometry
FITC anti-mouse/human Granzyme B	1:100	GB11	BioLegend	515403	Flow Cytometry
Pacific Blue anti-mouse/human Granzyme B	1:100	GB11	BioLegend	515408	Flow Cytometry
APC anti-human CD4	1:100	OKT4	BioLegend	317416	Flow Cytometry
PE/Cyanine 7 anti-human CD4	1:100	A161A1	BioLegend	357410	Flow Cytometry
FITC anti-human CD45RA	1:100	HI100	BioLegend	983002	Flow Cytometry
APC/Cyanine7 anti-human CD62L	1:100	DREG-56	BioLegend	304814	Flow Cytometry
FITC anti-human CD69	1:100	FN50	BioLegend	310904	Flow Cytometry
PerCP-Cyanine5.5 anti-human CD69	1:100	FN50	BioLegend	310926	Flow Cytometry
APC anti-human CD103	1:100	Ber-ACT8	BioLegend	350216	Flow Cytometry
Brilliant Violet 421 anti-human CD122	1:100	TU27	BioLegend	339010	Flow Cytometry
FITC anti-human HLA DR	1:100	L243	BioLegend	307632	Flow Cytometry
FITC anti-human IFN-γ	1:100	4S.B3	BioLegend	502506	Flow Cytometry
PerCP-Cy5.5 anti-human IL-2	1:100	MQ1-17H12	BioLegend	500322	Flow Cytometry

PE/Cyanine7 anti-human TNF-α	1:100	MAb11	BioLegend	502930	Flow Cytometry
FITC anti-mouse Ig λ1 λ2 λ3 light chain	1:100	R26-46	BD Biosciences	553434	Flow Cytometry
FITC anti-mouse IgG2a	1:100	R19-15	BD Biosciences	553390	Flow Cytometry
FITC anti-hamster IgG	1:100	G94-56	BD Biosciences	554008	Flow Cytometry
FITC anti-hamster IgG	1:100	G192-1	BD Biosciences	554026	Flow Cytometry

Supplementary Table 2. HLA DR monomer and HA1.7 construct sequences

DRα	MAISGVPVLGFFIIAVLMSAQESWAIKEEHVIIQAEFYLNPDQSGEFMFDFDGDEIFHVDMAKKETVWRLEEFGRFASFEAQGALANIAVDKANLEIMTKRSNYTPITNVPPEVTVLTNSPVELREPNVLICFIDKFTPPVVNVTWLRNGKPVTTGVSETVFLPREDHLFRKFHYLPFLPSTEDVYDCRVEHWGLDEPLLKHWEFDAPSPLPETTEVDGGGGGLTDTLQAETDQLEDEKSALQTEIANLLKEKEKLEFILAAGGSGGSGLNDIFEAQKIEWHEHHHHHH
DR1 β	MVCLKLPGGSCMTALTVTLMVLSSPLALAGDTGLPVSKMRMATPLLMQASGGGSLVPRGSGGGGSGDTRPRFLWQLKFECHFFNGTERVRLLERCIYNQEESVRFDSDVGEYRAVTELGRPDAEYWNSQKDLLEQRRAAVDTYCRHNYGVGESFTVQRRVEPKVTVYPSKTQPLQHHNLLVCSVSGFYPGSIEVRWFRNGQEEKAGVVSTGLIQNGDWTFQTLVMLETVPRSGEVYTCQVEHPSVTSPLTVEWRARSESAQSKVDGGGGGRIARLEEKVKTLKAQNSELASTANMLREQVAQLKQKVMNH
DR4 β	MVCLKLPGGSCMTALTVTLMVLSSPLALAGDTGLPVSKMRMATPLLMQASGGGSLVPRGSGGGGSGDTRPRFLEQVKHECHFFNGTERVRFLDRYFYHQEEYVRFDSDVGEYRAVTELGRPDAEYWNSQKDLLEQKRAAVDTYCRHNYGVGESFTVQRRVYPEVTVYPAKTQPLQHHNLLVCSVNGFYPGSIEVRWFRNGQEEKTGVVSTGLIQNGDWTFQTLVMLETVPRSGEVYTCQVEHPSLTSPLTVEWRARSESAQSKVDGGGGGRIARLEEKVKTLKAQNSELASTANMLREQVAQLKQKVMNH
HA1.7	MLLLLVPVLEVIFTLGGTRAQSVTQLGSHVSVSEGALVLLRCNYSSSVPPYLFWYVQYPNQGLQLLLKYTSAATLVKGINGFEAEFKKSETSFHLTKPSAHMSDAAEYFCAVSESPFGNEKLTFGTGTRLTIIPIQNPEPAVYQLKDPRSQDSTLCLFTDFDSQINVPKTMESGTFITDKTVLDMKAMDSKSNGAIAWSNQTSFTCQDIFKETNATYPSSDVPCDATLTEKSFETDMNLNFQNLSVMGLRILLLKVAGFNLLMTLRLWSSRRKRGSGATNFSLLKQAGDVEENPGPMGIRLLCRVAFCFLAVGLVDVKVTQSSRYLVKRTGEKVFLECVQDMDHENMFWYRQDPGLGLRLIYFSYDVKMKEKGDIPEGYSVSREKKERFSLILESASTNQTSMYLCASSSTGLPYGYTFGSGTRLTVVEDLRNVTPPKVSLFEPSKAEIANKQKATLVCLARGFFPDHVELSWWVNGKEVHSGVSTDPQAYKESNYSYCLSSRLRVSATFWHNPRNHFRCQVQFHGLSEEDKWPEGSPKPVTQNISAEAWGRADCGITSASYHQGVLSATILYEILLGKATLYAVLVSGLVLMAMVKKKNS
