# Supplementary material for: Nanoparticle-based modulation of CD4+ T cell effector and helper functions enhances adoptive immunotherapy
Source: Nat Commun. 2022 Oct 14;13:6086. doi: 10.1038/s41467-022-33597-y (PMC9568616; doi:10.1038/s41467-022-33597-y)
Supplement: Supplementary file 2 — Reporting Summary [file 41467_2022_33597_MOESM2_ESM.pdf]

Corresponding author(s): Jonathan Schneck
Last updated by author(s): Sep 4, 2022

Reporting Summary

Nature Portfolio wishes to improve the reproducibility of the work that we publish. This form provides structure for consistency and transparency in reporting. For further information on Nature Portfolio policies, see our Editorial Policies and the Editorial Policy Checklist.

Statistics

For all statistical analyses, confirm that the following items are present in the figure legend, table legend, main text, or Methods section.

n/a	Confirmed	
☐	☒	The exact sample size (n) for each experimental group/condition, given as a discrete number and unit of measurement
☐	☒	A statement on whether measurements were taken from distinct samples or whether the same sample was measured repeatedly
☐	☒	The statistical test(s) used AND whether they are one- or two-sided<br>*Only common tests should be described solely by name; describe more complex techniques in the Methods section.*
☐	☒	A description of all covariates tested
☐	☒	A description of any assumptions or corrections, such as tests of normality and adjustment for multiple comparisons
☐	☒	A full description of the statistical parameters including central tendency (e.g. means) or other basic estimates (e.g. regression coefficient) AND variation (e.g. standard deviation) or associated estimates of uncertainty (e.g. confidence intervals)
☐	☒	For null hypothesis testing, the test statistic (e.g. F, t, r) with confidence intervals, effect sizes, degrees of freedom and P value noted<br>*Give P values as exact values whenever suitable.*
☒	☐	For Bayesian analysis, information on the choice of priors and Markov chain Monte Carlo settings
☒	☐	For hierarchical and complex designs, identification of the appropriate level for tests and full reporting of outcomes
☐	☒	Estimates of effect sizes (e.g. Cohen's d, Pearson's r), indicating how they were calculated

Our web collection on statistics for biologists contains articles on many of the points above.

Software and code

Policy information about availability of computer code

Data collection	Sample size for in vivo experiments was calculated using G*Power v3.1.<br>Flow cytometry data was collected using CellQuest v3.3 and Attune NxT Software v4.2.0.<br>Image data was collected with iBright Analysis Software v4.0.1 and Zen 2.<br>Particle absorbance and flourescence data were collected with AD LD Analysis Software v1.2 and SkanIt RE v6.0.1.
Data analysis	Flow cytometry data was analyzed in FlowJo v10.7.1.<br>Image analysis was performed in ImageJ v2.1.0 and CellProfiler v4.0.7.<br>Data processing and statistical testing were performed in Microsoft Excel 2016 and GraphPad Prism v8.4.3.

For manuscripts utilizing custom algorithms or software that are central to the research but not yet described in published literature, software must be made available to editors and reviewers. We strongly encourage code deposition in a community repository (e.g. GitHub). See the Nature Portfolio guidelines for submitting code & software for further information.

Data

Policy information about availability of data

All manuscripts must include a data availability statement. This statement should provide the following information, where applicable:

- Accession codes, unique identifiers, or web links for publicly available datasets
- A description of any restrictions on data availability
- For clinical datasets or third party data, please ensure that the statement adheres to our policy

Raw data used for figures and plots are provided along with this paper as a Source Data file. Any additional datasets generated and/or analyzed for this manuscript are available from the corresponding author upon reasonable request.

Human research participants

Policy information about studies involving human research participants and Sex and Gender in Research.

Reporting on sex and gender	Information about sex of participants has been provided in the Human Studies section
Population characteristics	Volunteers used in this study included an Asian male, age 27, a Caucasian female, age 32, and an African American male, age 55.
Recruitment	Advertisements were posted throughout the university to recruit potential donors. On campus advertising may have skewed the demographics for this study; however, this is unlikely to have caused self-selection bias, due to the nature of this work.
Ethics oversight	The Johns Hopkins University Institutional Review Board approved these protocols.

Note that full information on the approval of the study protocol must also be provided in the manuscript.

Field-specific reporting

Please select the one below that is the best fit for your research. If you are not sure, read the appropriate sections before making your selection.

☒ Life sciences ☐ Behavioural & social sciences ☐ Ecological, evolutionary & environmental sciences

For a reference copy of the document with all sections, see nature.com/documents/nr-reporting-summary-flat.pdf

Life sciences study design

All studies must disclose on these points even when the disclosure is negative.

Sample size	Sample sizes for both in vitro and in vivo studies were determined through power analyses in G*Power v3.1, based on data from similar prior studies, using a power of 80% and a significance of 0.05
Data exclusions	Exclusion criteria, such as mice that had been fighting or were moribund due to other co-morbidities, were pre-established. In the in vivo tumor study, one animal was excluded from analysis as it was underweight and lethargic due to malocclusion.
Replication	All experiments, including in vitro and in vivo studies were conducted at least two times and could be reliably reproduced.
Randomization	Animals for all in vitro and in vivo studies were randomized and divided amongst experimental groups.
Blinding	All efforts were made to keep investigators blinded for animal studies. One exception needed to be made when an animal from an in vivo study needed to excluded due to malocclusion. At that point the investigators wer temporarily unblinded.

Reporting for specific materials, systems and methods

We require information from authors about some types of materials, experimental systems and methods used in many studies. Here, indicate whether each material, system or method listed is relevant to your study. If you are not sure if a list item applies to your research, read the appropriate section before selecting a response.

Materials & experimental systems

n/a	Involved in the study
[ ]	[x] Antibodies
[ ]	[x] Eukaryotic cell lines
[x]	[ ] Palaeontology and archaeology
[ ]	[x] Animals and other organisms
[x]	[ ] Clinical data
[x]	[ ] Dual use research of concern

Methods

n/a	Involved in the study
[x]	[ ] ChIP-seq
[ ]	[x] Flow cytometry
[x]	[ ] MRI-based neuroimaging

Antibodies

Antibodies used

Unlabeled antibodies: anti-mouse CD3e clone 145-2C11 (BioXcell, cat. BE0001-1), anti-mouse CD28 clone 37.51 (BioXcell, cat. BE0015-1), anti-mouse OX40 clone OX-86 (BioXcell, cat. BE0031), anti-mouse IFNγR clone GR-20 (BioXcell, cat. BE0029), anti-mouse I-A/I-E clone M5/114 (BioXcell, cat. BE0108), anti-mouse TNFα clone XT3.11 (BioXcell, cat. BE0058), and anti-mouse IL-10 clone JES5-2A5 (BioXcell, cat. BE0049), anti-human CD3 clone OKT-3 (BioXcell, cat. BE0001-2), anti-human CD28 clone 9.3 (BioXcell, cat. BE0248). Antibody dilutions for each of these is directly indicated in the manuscript.

Flourescent antibodies: PE anti-mouse CD3 clone 17A2 (BioLegend, cat. 100206), APC anti-mouse CD4 clone GK1.5 (BioLegend, cat. 100412), PerCP anti-mouse CD4 clone RM4-5 (BioLegend, cat. 100538), PE anti-mouse CD4 clone H129.19 (BioLegend, cat. 130310), APC-Cyanine7 anti-mouse CD4 clone GK1.5 (BioLegend, cat. 100414), APC anti-mouse CD8a clone 53-6.7 (BioLegend, cat. 100712), PerCP anti-mouse CD8 clone 53-6.7 (Biolegend, cat. 100732), APC/Cyanine 7 anti-mouse CD8a clone 53-6.7 (BioLegend, cat. 100714), PE/Cyanine7 anti-mouse CD8 clone 53-6.7 (BD Biosciences, cat. 561097), PerCP-Cy5.5 anti-mouse CD44 clone IM7 (BioLegend, cat. 103032), PE anti-mouse CD45.1 clone A20 (Biolegend, cat. 110708), PE anti-mouse CD45.2 clone 104 (BioLegend, cat. 109808), APC anti-mouse CD62L clone MEL-14 (BioLegend, cat. 104412), Alexa Fluor® 488 anti-mouse CD127 clone A7R34 (BioLegend, cat. 135018), PE/Cyanine7 anti-mouse CD197 (CCR7) clone 4B12 (BioLegend, cat. 120124), Brilliant Violet 605™ anti-mouse/human KLRG1 clone 2F1/KLRG1 (BioLegend, cat. 138419), FITC anti-mouse I-A/I-E clone M5/114.15.2 (BioLegend, cat. 107606), PE/Cyanine7 anti-mouse I-A/I-E clone M5/114.15.2 (BioLegend, cat. 107630), APC anti-mouse TCR β chain clone H57-597 (BioLegend, cat. 109212), FITC anti-mouse Foxp3 clone FJK-16s (eBioscience™, cat. 14-5773-82), PerCp-Cyanine5.5 anti-mouse/human T-bet clone eBio4B10 (eBioscience™, cat. 45-5825-82), APC anti-mouse/human RORγT clone AFKJS-9 (eBioscience™, cat. 17-6988-82), PE/Cyanine7 anti-mouse/human Gata3 clone TWAJ (eBioscience™, cat. 25-9966-42), APC anti-mouse IFN-γ clone XMG1.2 (BioLegend, cat. 505810), PE/Cyanine7 anti-mouse TNF-α clone MP6-XT22 (BioLegend, cat. 506324), PE anti-mouse IL-2 clone JES6-5H4 (BioLegend, cat. 503808), FITC anti-mouse/human Granzyme B clone GB11 (BioLegend, cat. 515403), Pacific Blue anti-mouse/human Granzyme B clone GB11 (BioLegend, cat. 515408), APC anti-human CD4 clone OKT4 (BioLegend, cat. 317416), PE/Cyanine 7 anti-human CD4 clone A161A1 (BioLegend, cat. 357410), FITC anti-human CD45RA clone HI100 (BioLegend, cat. 983002), APC/Cyanine7 anti-human CD62L clone DREG-56 (BioLegend, cat. 304814), FITC anti-human CD69 clone FN50 (BioLegend, cat. 310904), PerCP-Cyanine5.5 anti-human CD69 clone FN50 (BioLegend, cat. 310926), APC anti-human CD103 clone Ber-ACT8 (BioLegend, cat. 350216), Brilliant Violet 421™ anti-human CD122 clone TU27 (BioLegend, cat. 339010), FITC anti-human HLA DR clone L243 (BioLegend, cat. 307632), FITC anti-human IFN-γ clone 4S.B3 (BioLegend, cat. 502506), PerCP-Cy5.5 anti-human IL-2 clone MQ1-17H12 (BioLegend, cat. 500322), PE/Cyanine7 anti-human TNF-α clone MAb11 (BioLegend, cat. 502930), FITC anti-mouse Ig λ1 λ2 λ3 light chain clone R26-46 (BD Biosciences, cat. 553434), FITC anti-mouse IgG2a clone R19-15 (BD Biosciences, cat. 553390), FITC anti-hamster IgG clone G94-56 (BD Biosciences, cat. 554008), FITC anti-hamster IgG clone G192-1 (BD Biosciences, cat. 554026). All antibodies were used at a 1:100 dilution.

Validation

All antibodies used in this study contain validation statements on the Manufacturer's website. We further validated antibody specificity through use of proper negative and isotype controls, as indicated throughout the manuscript.

Eukaryotic cell lines

Policy information about cell lines and Sex and Gender in Research

Cell line source(s)

B16-SIY was a gift from Thomas Gajewski (The University of Chicago, IL, USA) (Kline, 2008), B16-F10 (ATCC no. CRL-6475) was a gift from Charles Drake (Johns Hopkins University, MD, USA), and B16-OVA was a gift from Jonathan Powell (Johns Hopkins University, MD, USA) (Falo, 1995). Lymphoblastoid Cell Lines (LCL) were a gift from the Johns Hopkins Human Immunogenetics Laboratory (Johns Hopkins University, MD, USA). Human Jurkat T cells clone E6-1 (ATCC no. TIB-152) and HEK-293F (Thermo Invitrogen no. R79007) were a gift from Jamie Spangler (Johns Hopkins University, MD, USA).

Authentication

Cell lines were not authenticated.

Mycoplasma contamination

Cell lines were not tested for mycoplasma contamination.

Commonly misidentified lines
(See ICLAC register)

None such cell lines were used.

Animals and other research organisms

Policy information about studies involving animals; ARRIVE guidelines recommended for reporting animal research, and Sex and Gender in Research

Laboratory animals

Similar numbers of 8-12 week old male and female mice were used for experiments. Specific strains included: C57BL/6 (B6) (Jackson,

	Strain # 000664), CD45.1 B6 (Jackson, Strain # 002014), OT-II (Jackson, Strain # 004194), SMART-A1 (Jackson Strain # 030450), OT-I Rag2-/- (Taconic, Strain # 2334), PMEL (Jackson, Strain# 005023), and 2C (Sha, 1988). Mice were housed in a specific pathogen free animal facility on a 12 light/12 dark light cycle, 65-75F, and 40-60% humidity. Experimental and control animals were co-housed.
Wild animals	No wild animals were used in this study.
Reporting on sex	While the precise number of male and female mice has not been collected, we performed all studies interchangeably with both male and female mice, yielding similar results.
Field-collected samples	No field collected animals were used in this study.
Ethics oversight	This study was approved by the Johns Hopkins University's Animal Care and Use Committee.

Note that full information on the approval of the study protocol must also be provided in the manuscript.

Flow Cytometry

Plots

Confirm that:

- [x] The axis labels state the marker and fluorochrome used (e.g. CD4-FITC).
- [x] The axis scales are clearly visible. Include numbers along axes only for bottom left plot of group (a 'group' is an analysis of identical markers).
- [x] All plots are contour plots with outliers or pseudocolor plots.
- [x] A numerical value for number of cells or percentage (with statistics) is provided.

Methodology

Sample preparation	Murine T cells were isolated by processing spleens and lymph nodes through a 70 µm cell strainer and then using no touch CD4+ T cell or CD8+ T cell isolation kits (Miltenyi). Human PBMC were isolated from healthy donor blood draws by Ficoll-Paque PLUS (GE Healthcare) density gradient centrifugation. PBMC were cryopreserved in a 90% FBS, 10% DMSO solution and stored in liquid nitrogen. Prior to use, cryopreserved PBMC were thawed with 50 U/mL benzonase Nuclease HC (EMD Millipore), washed, and then incubated overnight in T cell culture medium at 37°C. The following morning, CD4+ T cells were purified using no-touch CD4+ T cell isolation kits (Miltenyi).
Instrument	BD FACSCalibur (2-Laser) and Attune NxT (4-Laser)
Software	FlowJo v10.7.1.
Cell population abundance	No cell sorting was employed
Gating strategy	The main cell population was first gated by FSC-A/SSC-A to exclude debris, then by FSC-A/FSC-H to exclude doublets, and then by Live/Dead (Invitrogen) to exclude dead cells. Additional subsets were gated based on staining with antibodies (e.g. CD3, CD4, CD8, CD45.1, CD45.2), fluorescent dyes (e.g. CFSE, CTV, CellTrace Far Red), or tetramers/MHC-Ig dimers. Positivity thresholds were determined based on negative, isotype, non-cognate, or mock-treated controls as indicated throughout the manuscript.

- [x] Tick this box to confirm that a figure exemplifying the gating strategy is provided in the Supplementary Information.
